# Risk of heart failure in inflammatory bowel disease: a Swedish population-based study

**DOI:** 10.1093/eurheartj/ehae338

**Published:** 2024-05-21

**Authors:** Jiangwei Sun, Jialu Yao, Ola Olén, Jonas Halfvarson, David Bergman, Fahim Ebrahimi, Annika Rosengren, Johan Sundström, Jonas F Ludvigsson

**Affiliations:** Department of Medical Epidemiology and Biostatistics, Karolinska Institutet, Stockholm, Sweden; Department of Medical Epidemiology and Biostatistics, Karolinska Institutet, Stockholm, Sweden; Clinical Epidemiology Division, Department of Medicine Solna, Karolinska Institutet, Stockholm, Sweden; Sachs’ Children and Youth Hospital, Stockholm South General Hospital, Stockholm, Sweden; Department of Clinical Science and Education Södersjukhuset, Karolinska Institutet, Stockholm, Sweden; Department of Gastroenterology, Faculty of Medicine and Health, Örebro University, Örebro, Sweden; Department of Medical Epidemiology and Biostatistics, Karolinska Institutet, Stockholm, Sweden; Department of Medical Epidemiology and Biostatistics, Karolinska Institutet, Stockholm, Sweden; Department of Gastroenterology and Hepatology, Clarunis University Center for Gastrointestinal and Liver Diseases, Basel, Switzerland; Department of Molecular and Clinical Medicine, Institute of Medicine, Sahlgrenska Academy, University of Gothenburg, Gothenburg, Sweden; Sahlgrenska University Hospital VG-Region, Gothenburg, Sweden; Department of Medical Sciences, Uppsala University, Uppsala, Sweden; The George Institute for Global Health, University of New South Wales, Sydney, Australia; Department of Medical Epidemiology and Biostatistics, Karolinska Institutet, Stockholm, Sweden; Department of Pediatrics, Örebro University Hospital, Örebro, Sweden; Division of Digestive and Liver Disease, Department of Medicine, Columbia University Medical Center, New York, New York State, USA

**Keywords:** Inflammatory bowel disease, Heart failure, Nationwide, Cohort

## Abstract

**Background and Aims:**

Dysregulation of inflammatory and immune responses has been implicated in the pathogenesis of heart failure (HF). But even if inflammation is a prerequisite for inflammatory bowel disease (IBD), little is known about HF risk in IBD.

**Methods:**

In this Swedish nationwide cohort, patients with biopsy-confirmed IBD were identified between 1969 and 2017 [*n* = 81 749, Crohn’s disease (CD, *n* = 24 303), ulcerative colitis (UC, *n* = 45 709), and IBD-unclassified (IBD-U, *n* = 11 737)]. Each patient was matched with up to five general population reference individuals (*n* = 382 190) and IBD-free full siblings (*n* = 95 239) and followed until 31 December 2019. Flexible parametric survival models estimated the adjusted hazard ratio (aHR) and standardized cumulative incidence for HF, with 95% confidence intervals (CI).

**Results:**

There were 5582 incident HF identified in IBD patients (incidence rate [IR]: 50.3/10 000 person-years) and 20 343 in reference individuals (IR: 37.9) during a median follow-up of 12.4 years. IBD patients had a higher risk of HF than reference individuals (aHR 1.19, 95% CI 1.15–1.23). This increased risk remained significant ≥20 years after IBD diagnosis, leading to one extra HF case per 130 IBD patients until then. The increased risk was also observed across IBD subtypes: CD (IR: 46.9 vs. 34.4; aHR 1.28 [1.20–1.36]), UC (IR: 50.1 vs. 39.7; aHR 1.14 [1.09–1.19]), and IBD-U (IR: 60.9 vs. 39.0; aHR 1.28 [1.16–1.42]). Sibling-controlled analyses showed slightly attenuated association (IBD: aHR 1.10 [1.03–1.19]).

**Conclusions:**

Patients with IBD had a moderately higher risk of developing HF for ≥20 years after IBD diagnosis than the general population.

## Structured Graphical Abstract

**Graphical Abstract:**
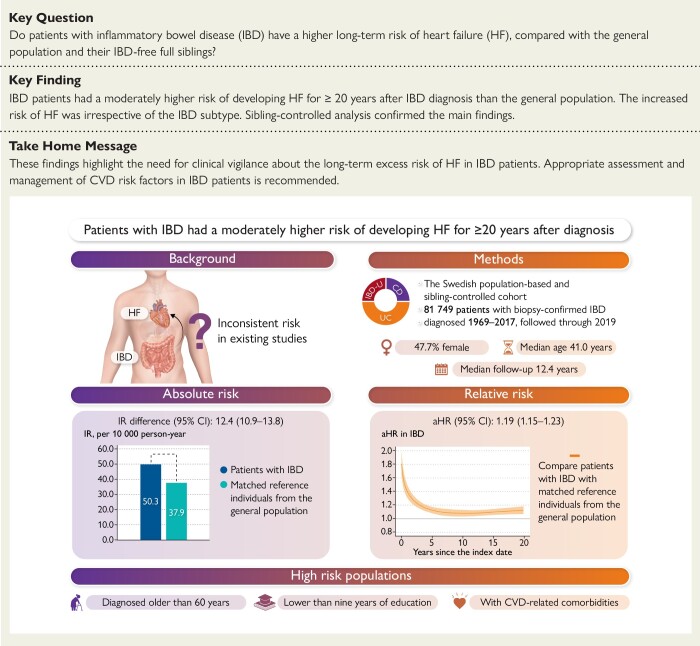
A long-term increased risk of heart failure in patients with inflammatory bowel disease. aHR, adjusted hazard ratio; CD, Crohn's disease; CVD, cardiovascular disease; CI, confidence interval; HF, heart failure; IBD(-U), inflammatory bowel disease (-unclassified); IR, incidence rate; UC, ulcerative colitis

**See the editorial comment for this article ‘Human immunology of heart failure: deconstructing inflammatory risk’, by M.J. Feinstein, https://doi.org/10.1093/eurheartj/ehae339.**

## Introduction

Inflammatory bowel disease (IBD), encompassing ulcerative colitis (UC), Crohn's disease (CD), and IBD-unclassified (IBD-U), is a chronic inflammatory disorder that primarily targets the gastrointestinal (GI) tract but often also has extraintestinal manifestations and complications.^1–3^ Studies have suggested a link between IBD and cardiovascular diseases (CVDs), including acute arterial events^4–7^ (i.e. ischaemic heart diseases, cerebrovascular disease, and peripheral artery diseases), venous thromboembolism,^8–10^ hypertension,^11^ and arrhythmias.^6,12,13^ These conditions precipitate heart failure (HF), a chronic syndrome resulting from structural and/or functional cardiac abnormalities, leading to elevated intracardiac pressure, or inadequate cardiac output.^14^ Although the incidence of HF has stabilized or even declined in industrialized countries, its prevalence is still increasing because of improved survival after HF diagnosis and ageing of the population.^15^ HF affects approximately 1%–3% of individuals globally, incurring high health care costs and with a one-year mortality of 15%–30%.^15^ Moreover, HF has been associated with intestinal congestion^16^ and IBD patients’ poor clinical outcomes (e.g. hospital readmissions and complications).^17^

Until now, research on HF risk in IBD has been inconsistent. Of three existing studies (summarized in Supplementary data online, *Table S1*), two reported an increased risk (1.37 in incidence rate ratio^18^ and 2.03 in hazard ratio [HR]^19^), while a recent US urban cohort study reported a null association.^20^ However, these studies had several limitations, including small sample size (e.g. observations in 736 IBD patients^19^ and 5078 IBD patients^20^), short median follow-up time (e.g. 6.4 years^18^ and 3.6 years^20^), inadequate control for important covariates, and data solely collected from one urban area.^20^ Moreover, earlier studies have not thoroughly explored HF in childhood-onset IBD. Such IBD tends to have a more severe phenotype and greater inflammatory burden than adult-onset IBD.^21^ Finally, earlier studies have failed to consider confounding from familial factors, which is an important limitation because both IBD and HF have a genetic predisposition.^14,22^

We conducted a nationwide population-based cohort study to investigate the long-term risk of HF in patients diagnosed with IBD between 1969 and 2017 and followed them until December 2019. Drawing upon the compelling evidence for the increased cardiovascular risk in IBD patients^4–13^ and the contributing role of inflammation in HF,^23,24^ we hypothesized that patients with IBD would be at an increased risk of HF. A sibling comparison design was also applied to control for shared familial factors.

## Methods

### Data source

This nationwide cohort was primarily based on the Swedish National Patient Register (NPR)^25^ and the Epidemiology Strengthened by histoPathology Reports in Sweden (ESPRESSO).^26^ The NPR covers inpatient care since 1964 (nationwide coverage from 1987) and specialized outpatient care since 2001.^25^ ESPRESSO is a histopathology cohort and collected GI biopsy reports from all 28 pathology departments in Sweden between 1965 and 2017,^26^ including date of biopsy, anatomic location, and morphology (by the adopted Swedish version of the Systematized Nomenclature of Medicine system).

### Identification of inflammatory bowel disease patients and comparison groups

IBD patients were identified as those receiving at least one International Classification of Diseases (ICD) code for IBD in the NPR and at least one IBD-indicative biopsy in the ESPRESSO (see Supplementary data online, *Table S2* for the definition of IBD). The index date (i.e. date of IBD diagnosis) was defined as the second date of receiving the first ICD code or the first IBD-indicative biopsy to avoid immortal time bias. Such a diagnostic approach has a high positive predictive value (PPV) of 95%–97%^27,28^ for IBD in Sweden. Different IBD phenotypes at index date, including CD location and perianal disease modifier, UC extent, and occurrence of primary sclerosing cholangitis as well as other extraintestinal manifestations were identified from the NPR, according to the Montreal classification criteria^29^ (see Supplementary data online, *Table S3* for ICD codes of IBD phenotypes).

Two comparison groups were used. Initially, each patient was randomly matched with up to five reference individuals from the Total Population Register (TPR)^30^ for year of birth, sex, county of residence, and calendar period. Next, full siblings of IBD patients were identified from the Swedish Multi-Generation Register.^31^ Reference individuals and full siblings had to live in Sweden and be free of IBD and HF at the date of matching (i.e. the index date). Individuals with heart transplant and congenital heart diseases were excluded from the analyses (see Supplementary data online, *Table S4A* for ICD codes).

### Follow-up and ascertainment of the outcomes

Each individual had a virtually complete follow-up using the Swedish personal identity number assigned to all residents across different registers. Follow-up started at the index date and ended with the diagnosis of HF, emigration, heart transplant, death, or 31 December 2019, whichever came first. In addition, individuals in the comparison groups were censored if they were diagnosed with IBD during follow-up. Incident HF was identified from the primary or secondary diagnoses in the NPR, where a validation study reported a PPV of 82%^32^ (see Supplementary data online, *Table S4A* for ICD codes).

### Covariates

In addition to the matching variables, we considered the following covariates. Country of birth (Nordic [Sweden, Denmark, Finland, Norway, and Iceland] or others) was identified from the TPR.^30^ Educational attainment (0–9, 10–12, ≥ 13 years, or ‘missing’), a proxy for socioeconomic status, was collected from the Swedish Longitudinal Integrated Database for Health Insurance and Labour Market Studies.^33^ Number of non-primary healthcare visits from two years to six months before the index date (0, 1, 2–3, and ≥ 4 times), a proxy for regular healthcare seeking behaviour, was retrieved from the NPR.^25^ Finally, from the NPR and the Prescribed Drug Register,^34^ we considered comorbidities before the index date, including ischaemic heart disease, arrhythmias, hypertension, anaemia, diabetes mellitus, obesity, dyslipidaemia, sleep problems, chronic kidney disease, chronic obstructive pulmonary disease (COPD, a proxy for smoking, only if the patient was diagnosed ≥40 years of age), and autoimmune diseases other than IBD (see Supplementary data online, *Table S4A* for their definitions).

### Statistical analyses

Flexible parametric survival models,^35^ which allow the effect of IBD to vary over time (time-varying effect) rather than being constant, were applied to calculate the adjusted HR (aHR) as well as standardized cumulative incidence of HF with 95% confidence intervals (CI). We explored the association for overall IBD and then for IBD subtypes (i.e. CD, UC, and IBD-U). In the population-based cohort, we conditioned our analyses on the matching variables (i.e. birth year, sex, county of residence, and calendar period) in model 1 and additionally adjusted for other covariates (i.e. country of birth, educational attainment, number of healthcare visits, and history of comorbidities) in model 2.

### Subgroup and sensitivity analyses

We calculated stratum-specific HRs by sex, age at index date (childhood onset: < 18 years, young adulthood onset: 18 to <40, middle-aged onset: 40 to <60, and elderly onset: ≥ 60; ), calendar period at index date (1969–89, 1990–2001, 2002–09, and 2010–17), educational attainment (0–9, 10–12, ≥ 13 years, or ‘missing’), number of healthcare visits between two years and six months before the index date (0, 1, 2–3, and ≥4), and history of comorbidities before the index date (for any CVD as well as separately for each comorbidity mentioned above, see Supplementary data online, *Table S4A*). To investigate the influence of disease phenotypes, we calculated phenotype-specific HRs by CD location, UC extent, and the occurrence of primary sclerosing cholangitis or other extraintestinal manifestations. To further explore any potential change in HF since the introduction of modern IBD therapy (e.g. biologics were first approved for IBD treatment in Sweden in 2002), we calculated the cumulative incidence of HF by calendar period at index date.

We conducted several sensitivity analyses to test the robustness of our results. First, we restricted the analysis to those with data on educational attainment (1990 onward). Second, because the Prescribed Drug Register was available only since July 2005, we restricted the analysis to those with an index date of January 2006 or later (to allow for 6 months of medication ascertainment) and further adjusted for cardiovascular medications prescribed before the index date (i.e. aspirin, non-aspirin antiplatelet medications, statins, non-statin lipid lowering medications, anticoagulants, antidiabetics, and antihypertensives, see Supplementary data online, *Table S4A* for the Anatomical Therapeutic Chemical codes). Third, to assess the potential influence of detection bias (i.e. work up for IBD increases the chance of diagnosing HF), surveillance bias (i.e. regular check-ups after IBD diagnosis increases the chance of early detection of HF), and reverse causation on the studied association, we repeated the main analysis by discarding the first 1 or 3 years of follow-up from the analysis. Fourth, to estimate the influence of HF definition, we identified patients with HF as those with at least two diagnoses. Fifth, to rule out the potential impacts of IBD treatments (including IBD-related surgery, steroids, and biologics, see Supplementary data online, *Table S4B* and *Table S4C* for their definitions) on the studied associations, we censored patients at exposure to these treatments. In the analysis for IBD surgery, we censored the follow-up at the date of first IBD-related surgery, in addition to the abovementioned censoring criteria. In the analysis for steroids and biologics, we limited the analysis to individuals with an index date of January 2006 or later and further censored the follow-up at the date of first steroid prescription or biological therapy after IBD diagnosis, respectively. Sixth, to investigate the potential influence of residual confounding from shared genetics and early environmental factors, we compared patients with IBD with their IBD-free full siblings, conditioning on family identifier and adjusting for birth year, sex, county of residence, calendar year, as well as the additional covariates in model 2 in the population-based cohort.

Finally, we described the comorbidities of incident HF at time of first diagnosis of HF in patients with IBD and their matched reference individuals. We considered the following comorbidities to be relevant (see Supplementary data online, *Table S4A* for their definitions): ischaemic heart disease, myocardial infarction, atrial fibrillation/flutter, hypertension, stroke, anaemia, dyslipidaemia, diabetes, obesity, chronic kidney diseases, and COPD.

Data analyses were conducted using software SAS (version 9.4; SAS Institute Inc, Cary, NC), Stata (version 16.1; StataCorp LP, College Station, TX), and R (version 3.6.0). A two-sided *P* ≤ .05 was considered statistically significant. This study is reported as per the Strengthening the Reporting of Observational Studies in Epidemiology (STROBE) guideline (see Supplementary data online).

## Results

We identified 81 749 patients with IBD (CD, 24 303; UC, 45 709; IBD-U, 11 737) and 382 190 matched reference individuals during the study period (*Table 1*). The median age at IBD diagnosis was 41.0 years and childhood-onset IBD comprised 8.5% of the patients. Of patients with IBD, 63% were diagnosed since 2002, 47.7% were female, and 92.0% were born in the Nordic countries. Patients with IBD had more healthcare visits and a higher prevalence of comorbidities than reference individuals. Colonic CD was found in 14.6% of CD patients, and extensive colitis in 15.6% of UC patients (see Supplementary data online, *Table S5*). More than 20% of study populations were followed for ≥20 years.

**Table 1:** Characteristics of patients with inflammatory bowel disease and their matched reference individuals, *n* (%)

	Reference individuals	Patients	Subtypes of IBD		
			CD	UC	IBD-U
*N*	382 190	81 749	24 303	45 709	11 737
Age at index date, years^a^					
Mean ± SD	41.6 ± 18.4	42.7 ± 18.9	40.4 ± 19.1	43.5 ± 18.4	44.5 ± 20.2
Median (IQR)	39.7 (26.5–55.4)	41.0 (27.2–57.2)	38.0 (24.4–55.0)	41.8 (28.7–57.4)	43.5 (27.6–60.7)
<18	34 521 (9.0)	6979 (8.5)	2828 (11.6)	3010 (6.6)	1141 (9.7)
18 to <40	158 239 (41.4)	32 486 (39.7)	10 047 (41.3)	18 323 (40.1)	4116 (35.1)
40 to <60	117 604 (30.8)	24 968 (30.5)	6912 (28.4)	14 613 (32.0)	3443 (29.3)
≥60	71 826 (18.8)	17 316 (21.2)	4516 (18.6)	9763 (21.4)	3037 (25.9)
Female	179 673 (47.0)	38 963 (47.7)	12 452 (51.2)	20 765 (45.4)	5746 (49.0)
Born in a Nordic country ^b^	338 980 (88.7)	75 207 (92.0)	22 086 (90.9)	42 445 (92.9)	10 676 (91.0)
Calendar period at index date^a^					
1969–1989	26 203 (6.9)	5432 (6.6)	2079 (8.6)	3033 (6.6)	320 (2.7)
1990–2001	117 097 (30.6)	24 807 (30.4)	7524 (31.0)	15 008 (32.8)	2275 (19.4)
2002–2009	122 641 (32.1)	26 260 (32.1)	7497 (30.9)	14 990 (32.8)	3773 (32.2)
2010–2017	116 249 (30.4)	25 250 (30.9)	7203 (29.6)	12 678 (27.7)	5369 (45.7)
Educational attainment, years					
0–9	82 195 (21.5)	17 939 (21.9)	5510 (22.7)	9783 (21.4)	2646 (22.5)
10–12	146 095 (38.2)	32 308 (39.5)	9269 (38.1)	18 439 (40.3)	4600 (39.2)
≥13	95 048 (24.9)	19 362 (23.7)	4951 (20.4)	11 536 (25.2)	2875 (24.5)
Missing	58 852 (15.4)	12 140 (14.9)	4573 (18.8)	5951 (13.0)	1616 (13.8)
Number of healthcare visits^c^					
0	288 389 (75.5)	48 339 (59.1)	13 805 (56.8)	28 615 (62.6)	5919 (50.4)
1	46 462 (12.2)	13 368 (16.4)	3962 (16.3)	7423 (16.2)	1983 (16.9)
2–3	29 253 (7.7)	10 625 (13.0)	3309 (13.6)	5469 (12.0)	1847 (15.7)
≥4	18 086 (4.7)	9417 (11.5)	3227 (13.3)	4202 (9.2)	1988 (16.9)
Disease history before index date^a,d^					
Any CVD	41 369 (10.8)	14 969 (18.3)	4111 (16.9)	7945 (17.4)	2913 (24.8)
Ischaemic heart disease	8979 (2.4)	3014 (3.7)	721 (3.0)	1673 (3.7)	620 (5.3)
Arrhythmias	5842 (1.5)	1803 (2.2)	466 (1.9)	929 (2.0)	408 (3.5)
Hypertension	14 226 (3.7)	5463 (6.7)	1499 (6.2)	2669 (5.8)	1295 (11.0)
Anaemia	1591 (0.4)	2834 (3.5)	1249 (5.1)	995 (2.2)	590 (5.0)
Diabetes	8399 (2.2)	2798 (3.4)	708 (2.9)	1462 (3.2)	628 (5.4)
Obesity	3743 (1.0)	995 (1.2)	348 (1.4)	406 (0.9)	241 (2.1)
Dyslipidaemia	4201 (1.1)	1370 (1.7)	317 (1.3)	712 (1.6)	341 (2.9)
Sleep problems	4545 (1.2)	1312 (1.6)	392 (1.6)	637 (1.4)	283 (2.4)
Chronic kidney diseases	810 (0.2)	509 (0.6)	140 (0.6)	218 (0.5)	151 (1.3)
COPD^e^	2331 (0.6)	1198 (1.5)	387 (1.6)	543 (1.2)	268 (2.3)
Autoimmune diseases	25 832 (6.8)	10 567 (12.9)	3910 (16.1)	4491 (9.8)	2166 (18.5)
Follow-up time, years					
Median (IQR)	12.9 (7.3–19.0)	12.4 (6.9–18.8)	12.9 (7.1–19.6)	13.2 (7.4–18.9)	9.3 (5.3–14.9)
<1	6522 (1.7)	2201 (2.7)	636 (2.6)	1056 (2.3)	509 (4.3)
1–4	44 373 (11.6)	10 070 (12.3)	2864 (11.8)	5007 (11.0)	2199 (18.7)
5–9	92 735 (24.3)	20 132 (24.6)	5805 (23.9)	10 683 (23.4)	3644 (31.1)
10–19	152 811 (40.0)	32 282 (39.5)	9206 (37.9)	19 035 (41.6)	4041 (34.4)
≥20	85 749 (22.4)	17 064 (20.9)	5792 (23.8)	9928 (21.7)	1344 (11.5)

Abbreviations: CD, Crohn’s disease; COPD, chronic obstructive pulmonary disease; CVD, cardiovascular diseases; IBD-U, inflammatory bowel disease unclassified; IQR, interquartile range; SD, standard deviation; UC, ulcerative colitis.

^a^Index date: date of IBD diagnosis for patients, and date of selection for their matched population reference individuals.

^b^Sweden, Denmark, Finland, Norway, and Iceland.

^c^Defined as the number of healthcare visits between 2 years and 6 months before the index date.

^d^See Supplementary data online, *Table S4* for diseases’ definitions.

^e^Only if the patient was diagnosed ≥ 40 years of age.

### Inflammatory bowel disease and risk of heart failure

During a median follow-up of 12.4 years, 5582 IBD patients were diagnosed with HF (incidence rate [IR]: 50.3/10 000 person-years), compared with 20 343 (IR: 37.9) in reference individuals (*Table 2*). The IR difference was 12.4 (46.9 vs. 34.4) for CD, 10.4 (50.1 vs. 39.7) for UC, and 21.9 (60.9 vs. 39.0) for IBD-U.

**Table 2:** Incident heart failure in patients with inflammatory bowel disease and their matched reference individuals

	No. of events (IR, per 10 000 person-years)		IR difference (95%CI), per 10 000 person-years	HR (95%CI)	
	Patients	References		Model 1^a^	Model 2^b^
Overall IBD	5582 (50.3)	20 343 (37.9)	12.4 (10.9–13.8)	1.36 (1.31–1.40)	1.19 (1.15–1.23)
CD	1614 (46.9)	5800 (34.4)	12.4 (10.0–14.9)	1.48 (1.39–1.57)	1.28 (1.20–1.36)
UC	3200 (50.1)	12 156 (39.7)	10.4 (8.5–12.3)	1.26 (1.21–1.32)	1.14 (1.09–1.19)
IBD-U	768 (60.9)	2387 (39.0)	21.9 (17.3–26.4)	1.57 (1.43–1.72)	1.28 (1.16–1.42)

Abbreviations: CD, Crohn's disease; CI, confidence interval; HR, hazard ratio; IBD-U, inflammatory bowel disease unclassified; IR, incidence rate; UC, ulcerative colitis.

^a^Conditioned on the matching variables (birth year, sex, county of residence, and calendar year).

^b^Further adjusted for country of birth, educational attainment, number of healthcare visits, ischemic heart disease, arrhythmias, hypertension, anaemia, dyslipidaemia, diabetes, obesity, sleep problems, chronic kidney diseases, chronic obstructive pulmonary disease (only if diagnosed ≥ 40 years), and autoimmune diseases.

**Figure 1:**
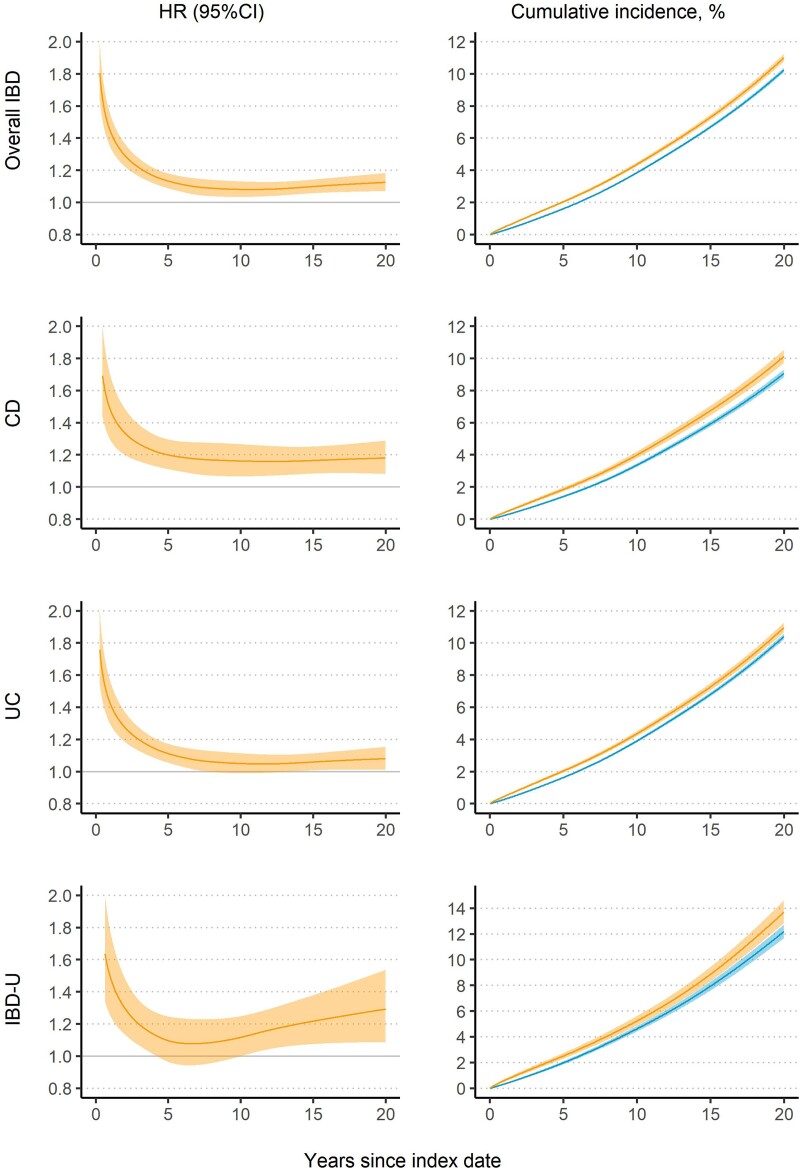
Hazard ratio (HR) and standardized cumulative incidence for heart failure in patients with inflammatory bowel disease (IBD) compared with their matched reference individuals, both with 95% confidence interval (CI). The adjusted flexible parametric survival model estimated HR and standardized cumulative incidence (upper line with 95%CI: patients with IBD; lower line with 95%CI: the matched reference individuals). CD, Crohn’s disease; IBD-U, IBD-unclassified; UC, ulcerative colitis

After multivariable adjustment, patients with IBD were at an increased risk of HF. The aHR was 1.19 (95%CI: 1.15–1.23) in overall IBD, 1.28 (1.20–1.36) in CD, 1.14 (1.09–1.19) in UC, and 1.28 (1.16–1.42) in IBD-U (*Table 2*). The relative risk of HF was highest initially and then decreased to a plateau from approximately 5–6 years while remaining significantly elevated even 20 years after IBD diagnosis (*Figure 1*). The cumulative incidence of HF was constantly higher in IBD patients, irrespective of subtype (*Figure 1*). The differences in 20-year cumulative incidence were 0.77% for IBD, 1.05% for CD, 0.56% for UC, and 1.52% for IBD-U, corresponding to one extra case of HF per 130 IBD patients, 95 CD patients, 179 UC patients, and 66 IBD-U patients.

### Subgroup and sensitivity analyses

**Figure 2:**
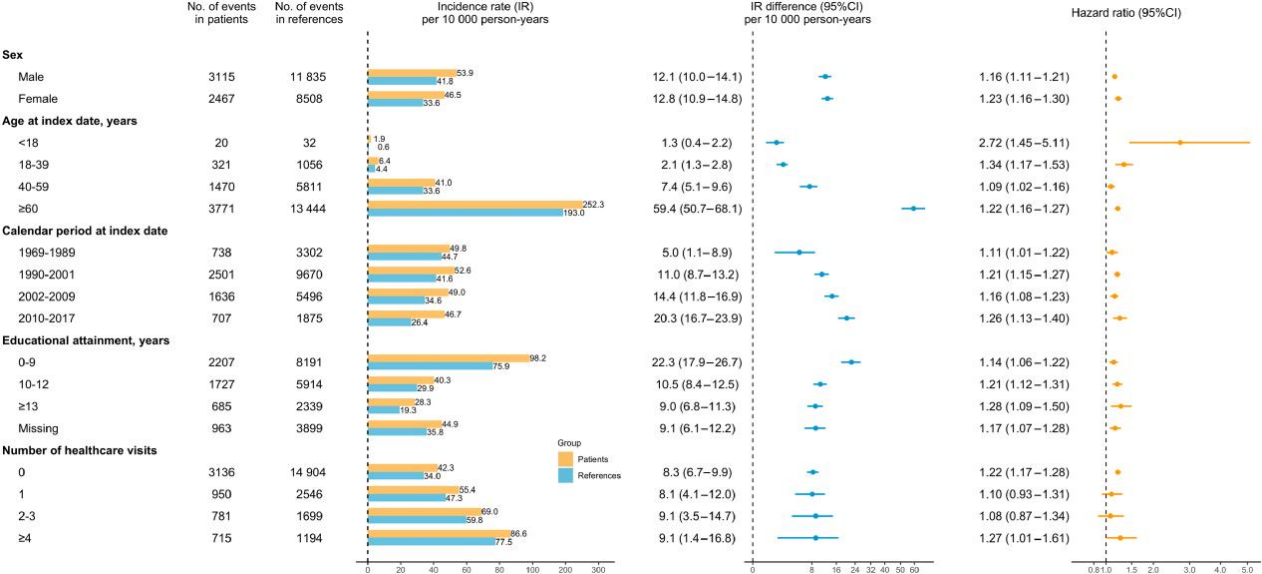
Incident heart failure in patients with inflammatory bowel disease compared with their matched reference individuals, subgroup by baseline (i.e. at the index date) characteristics. The hazard ratio was estimated from the adjusted flexible parametric survival model. CI, confidence interval; IR, incidence rate

Subgroup analyses by demographic characteristics revealed marked variations in the absolute risk of HF (*Figure 2*, Supplementary data online, *Table S6*). Specifically, absolute risks were highest in elderly onset IBD, patients with 0–9 years of education, and those with more healthcare visits during two years and six months before the index date. The IR difference of HF decreased with educational attainment but substantially increased with age and calendar period. Across calendar periods, a higher cumulative incidence of HF was consistently observed in IBD patients, and the introduction of modern IBD therapy since 2002 did not seem to have greatly influenced the cumulative incidence of HF (see Supplementary data online, *Figure S1*). However, for relative risk, it was highest in childhood-onset IBD (aHR = 2.72 [1.45–5.11], *P _for interaction_ <* .001) compared with other age groups, and was not modified by calendar period or educational attainment (both *P _for interaction_ >* .05). A higher relative risk was observed in females (aHR = 1.23 [1.16–1.30]) compared with males (aHR = 1.16 [1.11–1.21], *P _for interaction_ =* .0128). Similar patterns were also observed in patients with CD, UC, and IBD-U (see Supplementary data online, *Table S7*).

**Figure 3:**
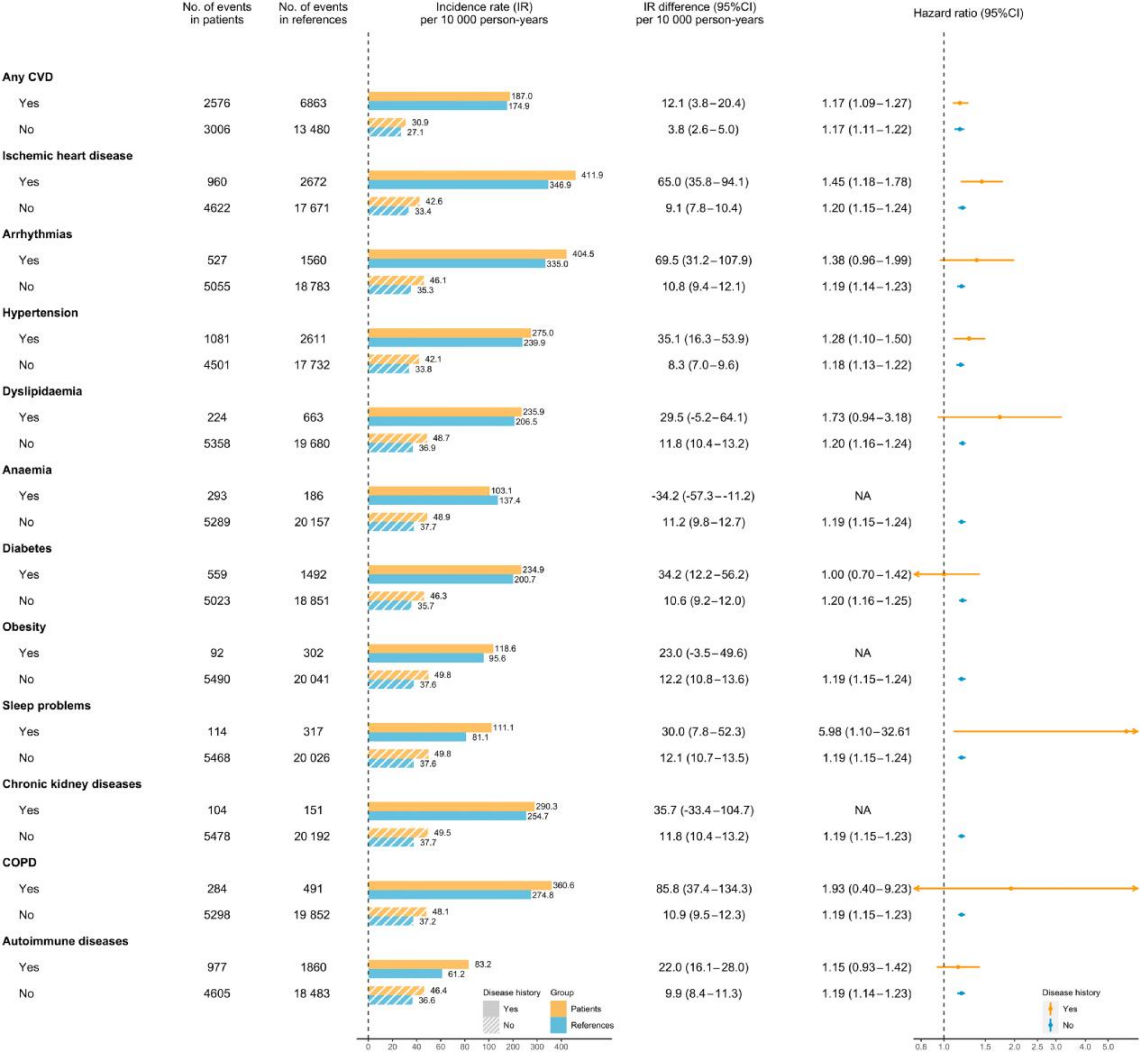
Incident heart failure in patients with inflammatory bowel disease compared with their matched reference individuals, subgroup by baseline (i.e. at the index date) disease histories. The hazard ratio was estimated from the adjusted flexible parametric survival model. CI, confidence interval; IR, incidence rate

Although cardiovascular-related comorbidities and autoimmune diseases did not substantially affect the relative risk elevations for HF, the absolute risk of HF was higher in IBD patients with these conditions (*Figure 3*).

Significant differences in terms of absolute and relative risks were not observed when stratifying the analysis by IBD phenotype identified at the index date (see Supplementary data online, *Table S8*). However, patients with other extraintestinal manifestations suffered from higher absolute risks and higher IR differences of HF, and the relative risk for UC patients with other extraintestinal manifestations was higher than for other phenotypes (aHR = 1.58 [1.17–2.13]) (see Supplementary data online, *Table S8*).

We observed robust associations in sensitivity analyses after restricting the analyses to those with educational data, including only those with an index date of January 2006 or later and further adjusting for cardiovascular medications, discarding the first one or three years of follow-up from the analyses, as well as defining HF as having at least two diagnoses (see Supplementary data online, *Table S9*). The positive association between IBD and HF remained even when patients with IBD were censored at date of IBD-related surgery (aHR = 1.19 [1.15–1.24]), steroid prescription (aHR = 1.19 [1.04–1.35]), and biological therapy (aHR = 1.19 [1.11–1.28]) (see Supplementary data online, *Table S9*).

### Sibling comparison

In the sibling-controlled cohort, we identified 52 761 IBD patients (15 771 CD, 29 567 UC, and 7423 IBD-U) with at least one IBD-free full sibling alive at the index date to address the potential residual confounding from shared familial factors (see Supplementary data online, *Table S10*). Compared with their IBD-free full siblings, IBD patients were younger and had a higher prevalence of comorbidities. In sibling comparison analyses, we also observed positive association between IBD and HF but the aHRs were slightly lower than those from the population-based cohort (aHR = 1.10 [1.03–1.19] for overall IBD, 1.15 [1.00–1.31] for CD, 1.06 [0.96–1.16] for UC, and 1.25 [1.02–1.53] for IBD-U) (see Supplementary data online, *Table S11*).

### Comorbidities before heart failure diagnosis

The mean age of HF diagnosis in IBD patients and their matched reference individuals was 74.8 and 75.9 years, respectively. Compared with incident HF patients in reference individuals, those with incident HF in IBD patients more often had records of ischaemic heart disease, hypertension, anaemia, diabetes, chronic kidney diseases, and COPD before the date of HF diagnosis (all *P*-values <.001) (see Supplementary data online, *Table S12*).

## Discussion

In this nationwide cohort study, we observed an increased risk of HF in patients with IBD. The relative risk was highest during the first 5–6 years and decreased thereafter but remained significantly elevated 20 years after IBD diagnosis, resulting in one extra case of HF per 130 patients with IBD. The relative risk was increased in both males and females, and across all age groups and calendar periods at IBD diagnosis. Notably, although the relative risk was more pronounced in childhood-onset IBD, the highest absolute risk was observed in elderly onset IBD. In addition, sibling comparison analysis showed that shared familial factors could not fully explain the associations (*Structured Graphical Abstract*).

We observed that patients with traditional CVD risk factors before IBD diagnosis (e.g. old age, low education attainment, and comorbidities^36,37^) had a higher absolute risk to develop HF. In every 100 person-years, there were 2.5 incident HF cases in elderly onset IBD (vs. 0.02 in childhood-onset IBD, *Figure 2*), 1.0 in patients with 0–9 years of education (vs. 0.3 in patients with ≥13 years of education, *Figure 2*), and 1.9 in patients with any concomitant CVD before IBD diagnosis (vs. 0.3 in patients without any concomitant CVD) (*Figure 3*).

### Comparison with earlier literature

To date, our study is the largest to investigate HF risk across IBD and all subtypes (i.e. CD, UC, and IBD-U) and suggests that patients with IBD were at an increased risk of HF (aHR = 1.19 [1.15–1.23] for overall IBD). Our slightly lower point estimate compared with findings from Kristensen et al. (IR 1.37, 95% CI: 1.26–1.49)^18^ and Aniwan et al. (aHR 2.03, 95% CI: 1.36–3.03)^19^ (summarized in Supplementary data online, *Table S1*), may have two explanations. First, the longer follow-up in our cohort compared with Kristensen et al. study (12.4 years vs. 6.4 years^18^) may lead to a lower average relative risk, given that the relative risk of HF decreased over follow-up time (as shown in *Figure 1*). Second, different adjustments for potential confounders in our study might yield a more conservative estimate, because we included a broader range of potential risk factors for HF (e.g. anaemia and sleep problems^14,38,39^).

The high relative risk of HF in childhood-onset IBD (aHR 2.72) aligned with previous findings in childhood-onset IBD for stroke,^7^ malignancy,^40,41^ and death,^42^ which could be attributed to the prominence of IBD in an age group (children) in which few individuals suffer from HF, but possible also to ascertainment bias because very few healthy children are evaluated for HF. In addition, more severe disease activity in childhood-onset patients may contribute to this finding. Compared with adult-onset IBD, younger patients tend to have more complicated disease courses^21^ and use more biologics and immunomodulators.^43^ Although HF in childhood-onset IBD remained much less common than in adult-onset or elderly onset IBD patients, we should note a poor prognosis in young patients with HF.^44^ For example, the estimated life-years lost was 20.1 years less than in the general population in Sweden if HF was diagnosed at age 40 years.^44^ We noted a gradual increase in the IR difference of HF between individuals with and without IBD over calendar periods. This trend may be influenced by varying follow-up durations across different calendar periods. For instance, reference individuals in the first calendar period had a longer follow-up time, reaching an age where HF is more common, compared with those in the most recent calendar period. The observed decrease in the absolute risk of HF with length of educational attainment may be partially due to the older age composition in the individuals with 0–9 years of education (median age: 49.8 vs. 40.4 in those with 10–12 years of education, and 39.9 in those with ≥ 13 years of education), which implied a higher prevalence of traditional CVD risk factors. Moreover, education attainment, together with other social factors (e.g. income and employment) can also greatly affect individuals’ health status.^45^ Previous evidence has also suggested that individuals with lower education attainment are more susceptible to medication non-adherence, suboptimal disease management, and worse outcomes, in patients with either IBD^46,47^ or CVD.^48,49^

Unlike Aniwan et al.,^19^ we did not observe an increased risk of HF in UC patients with extensive colitis. However, in a stratum of UC patients, other extraintestinal manifestations were associated with both a larger absolute risk and IR difference. Given that the absolute risk of HF in reference individuals in this stratum (IR: 35.2 per 10 000 person-years, Supplementary data online, *Table S8*) was similar to that in the main analysis (IR: 39.7 per 10 000 person-years, *Table 2*), we hypothesized that the findings in UC patients with other extraintestinal manifestations might be attributed to the underlying disease activity.^50^ However, more studies are needed to validate or reject this hypothesis.

Compared with the Aniwan et al. study,^19^ our sibling comparison analysis more accurately captured the familial disease histories of patients with IBD. While a slightly younger age in siblings compared with the reference individuals may have contributed to the shift in absolute risk excess, the relative risk was only slightly attenuated, implying that unmeasured confounding (e.g. genetics and early environmental factors shared within families) may partially but not fully explain our conclusions.^14,22^

### Potential mechanisms

Although underlying mechanisms driving an association between IBD and HF remains unclear, chronic systemic inflammation and microbiome alterations in IBD have been proposed.^23,24^ Elevated pro-inflammatory cytokines (e.g. tumour necrosis factor-α, interleukin [IL]-1, and IL-6) and lipopolysaccharides from altered microbiota promote endothelial dysfunction,^51^ in which signalling pathways that modulate myocardial hypertrophy, relaxation, and stiffness are impaired.^52^ The consequent hemodynamic stress may lead to adverse left ventricular remodelling and diastolic dysfunction, potentially resulting in HF.^24^ With the atherosclerotic and arrhythmogenic effect of both pro-inflammatory cytokines and lipopolysaccharides,^51^ patients with IBD are more likely to develop acute arterial events^4,7^ and arrhythmias,^13^ which may contribute to HF.^23^ Moreover, patients with IBD are more susceptible to anaemia,^53^ infection,^54^ surgery,^43^ and steroid therapy,^43^ which could also increase the risk of HF.

### Strengths and limitations

This nationwide population-based and sibling-controlled cohort had a virtually complete follow-up of >80 000 biopsy-confirmed IBD patients. It enabled precise assessment of the association across various subgroups and to investigate its temporal patterns over follow-up time. Additionally, the diagnostic accuracy for IBD (95% PPV for IBD^27^) reduced the risk of information bias. Moreover, our additional comparison with siblings offered an optimal context for alleviating unmeasured confounding by familiar factors.

Limitations of our study should also be noted. First, due to the absence of primary care data and the incomplete coverage of inpatient care (nationwide coverage since 1987) and outpatient (since 2001) in the NPR,^55^ some patients with IBD or HF may not have been identified. Moreover, the diagnostic accuracy for HF was only validated in the inpatient care with a PPV of 82%,^32^ but not among outpatient (where echocardiography is likely less often performed than in inpatient care), therefore the PPV of HF in our study may be lower than 82%. Second, we lacked detailed information on lifestyle protective and risk factors for HF that may confound the association (e.g. diet, physical activities, and smoking^14^) due to the register-based nature of this study. Because those factors tend to cluster within families, similar results from the sibling comparison analysis might have relieved such concern to some extent. However, given the genetic predisposition of IBD^22^ and HF,^14^ some siblings may have undiagnosed IBD or HF, which could also make the effect estimates in sibling analysis too conservative. Moreover, given a recent meta-analysis suggested that smoking was associated with CD (odds ratio 1.76 for current smoker) and inversely associated with UC (odds ratio 0.58 for current smoker),^56^ we performed a sensitivity analysis to investigate how sensitive our observed association between CD and HF (HR 1.28 in the population-based cohort) is to smoking. The result showed that for smoking to explain away the observed association between CD and HF, it would have to have both a stronger association with HF (i.e. HR >2.5^57^) and a highly imbalanced distribution between patients with CD and reference individuals (see Supplementary data online, *Figure S2*). Third, because of data unavailability, our analysis did not incorporate phenotypes of HF (e.g. HF with preserved ejection fraction [HFpEF] vs. HF with reduced ejection fraction [HFrEF]). However, we observed a greater burden of comorbidities in IBD patients with incident HF (including ischaemic heart disease, hypertension, anaemia, diabetes, chronic kidney diseases, and COPD), which are risk factors for cardiac dysfunction and HFpEF/HFrEF and more prevalent in HFpEF.^58,59^ Therefore, future studies are needed to ascertain whether HFpEF or HFrEF is the main subtype of HF in IBD patients who develop HF. Fourth, although we observed a positive association between IBD and HF in patients naïve to IBD surgery, steroids, or biologics, exploring the potential influences of IBD medications and disease activity/severity on HF was not within the scope of our study. Given that some IBD medications might have a cardio-protective effect (e.g. 5-aminosalicylic acid,^60^) while others may have an opposite effect (e.g. steroids^60^ and tofacitinib^61^), further studies that combine appropriate study design (e.g. a new user design) with solid statistical methods (e.g. propensity score matching/weighting^62^ or target trial emulation^63^) are needed to determine the extent to which the IBD and HF association is influenced by those factors and to disentangle effects of IBD medications from disease activity/severity. Finally, our study was observational and exclusively conducted in Sweden. Therefore, we do not claim causal relationship between IBD and HF and caution should be exercised when generalizing our results to different regions and ethnicities.

### Implications

The risk elevation for HF in IBD was moderate, with an absolute increase of 12.4/10 000 person-years and a relative increase of +19%. Given a persistently increased risk of HF even 20 years after IBD diagnosis, the impact of IBD is expected to escalate with the rising burden of cardiovascular risk factors when patients get older.^64^ Our findings have important implications. First, healthcare providers (e.g. cardiologists, gastroenterologist, and general practitioners) and patients themselves should be aware of the long-term increased risk of HF in patients with IBD, especially in patients over 60 years old, in those with less than 9 years of education, and in those with cardiovascular-related comorbidities before IBD diagnosis, which may help to identify high-risk groups at relatively early stage. For those high-risk groups, management of modifiable HF risk factors is recommended and can be done according to current guideline for HF risk prevention.^14,65,66^ Second, optimal antiinflammatory therapy aiming at remission for IBD but with less adverse cardiovascular effects should be carefully considered, especially when prescribing IBD medications with cardiovascular risks (e.g. steroids^60^ and tofacitinib^61^) to IBD patients with traditional HF risk factors. Third, our results, together with previous evidence of IBD on CVD, could be used for development of new guidelines on the assessment and management of CVD in patients with IBD.^67^

## Conclusions

In conclusion, patients with IBD (overall as well as CD, UC, and IBD-U) had a moderately higher risk of HF for ≥ 20 years after IBD diagnosis than the general population. In parallel with the rising prevalence of IBD^68^ and its unfavourable cardiovascular risk profile,^37^ the substantial burden of HF on individuals and society^15^ warrants attention for early identification and treatment in patients with IBD.

## Supplementary data

Supplementary data are available at *European Heart Journal* online.

## Supplementary Material

ehae338_Supplementary_Data

## Declarations

### Data Availability

The data set cannot be shared directly under current legislation for data protection and must be requested directly from the respective registry holders, Statistics Sweden (information@scb.se) and the Swedish National Board of Health and Welfare (registerservice@socialstyrelsen.se), after approval by the Swedish Ethical Review Authority.
