# Supplementary material for: Risk of heart failure in inflammatory bowel disease: a Swedish population-based study
Source: Eur Heart J. 2024 May 21;45(28):2493–504. doi: 10.1093/eurheartj/ehae338 (PMC11260193; doi:10.1093/eurheartj/ehae338)
Supplement: ehae338_Supplementary_Data [file ehae338_supplementary_data.doc]

**Supplementary material**

**Risk of heart failure in inflammatory bowel disease: a Swedish population-based study**

Sun et al.

Contents

[**Table S1** Previous population-based studies on the association between IBD and heart failure 3](#__RefHeading___Toc164173362)

[**Table S2** ICD codes and SNOMED codes defining IBD. 5](#__RefHeading___Toc164173363)

[**Table S3** ICD codes for each phenotype of CD, UC, and IBD-U 6](#__RefHeading___Toc164173364)

[**Table S4a** Definitions of the exclusion criteria, outcomes, comorbidities, and medications 7](#__RefHeading___Toc164173365)

[**Table S4b** IBD-related surgical procedure codes 11](#__RefHeading___Toc164173366)

[**Table S4c** IBD medications: biologics and steroids 13](#__RefHeading___Toc164173367)

[**Table S5** Clinical phenotypes of patients with IBD, n (%) 14](#__RefHeading___Toc164173368)

[**Table S6** Subgroup analyses of heart failure in patients with IBD and their matched reference individuals 15](#__RefHeading___Toc164173369)

[**Table S7** Subgroup analyses of heart failure in patients with CD, UC, IBD-U and their matched reference individuals 17](#__RefHeading___Toc164173370)

[**Table S8** Incident heart failure in patients with IBD and their matched reference individuals, stratified by the phenotypes of the Montreal Classification 18](#__RefHeading___Toc164173371)

[**Table S9** Sensitivity analyses of incident heart failure in patients with IBD and their matched reference individuals 19](#__RefHeading___Toc164173372)

[**Table S10** Characteristics of patients with IBD and their IBD-free full siblings, n (%) 21](#__RefHeading___Toc164173373)

[**Table S11** Incident heart failure in patients with IBD and their IBD-free full siblings 23](#__RefHeading___Toc164173374)

[**Table S12** Characteristics of incident heart failure at time of first diagnosis of heart failure in patients with IBD and their matched reference individuals 24](#__RefHeading___Toc164173375)

[**Figure S1** Standardized cumulative incidence with 95% confidence interval (CI) for heart failure, stratified by calendar period at index date. 25](#__RefHeading___Toc164173376)

[**Figure S2** Sensitivity analysis of unmeasured confounding for the association of CD with heart failure due to smoking. 26](#__RefHeading___Toc164173377)

**Table S1** Previous population-based studies on the association between IBD and heart failure

| **First author, publication year,  place,  data source, study period, follow-up time** | **Sample size** | **Age at IBD diagnosis, years** | **Female, %** | **Number of outcomes in IBD** | **Incidence rate of heart failure, per 10,000 person years** | **First year of follow-up included?** | **Covariates** | **Main findings** |
| --- | --- | --- | --- | --- | --- | --- | --- | --- |
| **Current study** | | | | | | | | |
| Sun,  (current study), Sweden, Nationwide,  1969-2017 followed through 2019,  Median 12.4 years,  (IQR: 6.9-18.8) | IBD: **81,749 CD: 24,303 UC: 45,709 IBD-U: 11,737** | Median 41.0,  all ages included | 47.7 | IBD: 5,582  CD: 1,614  UC: 3,200  IBD-U: 768 | IBD: 50.3 vs 37.9  CD: 46.9 vs 34.4 UC: 50.1 vs 39.7 IBD-U: 60.9 vs 39.0 | Included, excluded in sensitivity analysis | (Model 1)  1. birth year  2. sex  3. county of residence  4. calendar year  (Model 2)  5. country of birth  6. educational attainment  7. number of healthcare visits  8. ischemic heart disease  9. arrhythmias  10. hypertension  11. anaemia  12. dyslipidaemia  13. diabetes  14. obesity  15. sleep problems  16. chronic kidney diseases  17. COPD  (diagnosed **≥**40 years)  18. autoimmune diseases. | Increased risk of heart failure in IBD.  IBD: aHR=1.19  (95% CI: 1.15-1.23)  CD: aHR=1.28  (1.20-1.36)  UC: aHR=1.14  (1.09-1.19)  IBD-U: aHR=1.28  (1.16-1.42).  Highest relative risk in childhood-onset IBD: aHR = 2.72 (1.45-5.11) |
| **Individual studies** | | | | | | | | |
| Prasada^1^,  2020, The US,  the Northwestern Medicine Enterprise Data Warehouse (single urban medical system),  2000/1/1-2019/1/1,  Median 3.6 years | IBD: 5,078 | Mean 44.55 (SD: 16.49) only adult included | 56.9 | IBD: 73 | IBD: 7.49 vs 5.98 | Excluded | (Model1)  1. age  2. sex  3. insurance status  4. baseline year  5. baseline hypertension  6. diabetes  (Model 2)  7. CKD  (Model 3)  8. baseline and interim coronary heart disease | No association between inflammatory bowel disease and incident heart failure  aHR= 0.95 (0.74-1.22) (Model 2) |
| Aniwan^2^,  2018, the US,  Regional (Olmsted County, Minnesota),  1980-2010,  Median: 14.3 years (IQR: 8.3-22.3) | IBD: 736  CD: 339  UC: 397 | Median (IQR):  IBD: 34.1 (24.0–47.8)  CD: 31.2 (21.8–47.4)  UC: 35.6 (25.0–48.1) all ages included | IBD: 45  CD: 48  UC: 43 | IBD: 105  CD: 51  UC: 54 | IBD: 45.5 vs 29.1  CD: 47.8 vs 32.2  UC: 43.6 vs 26.5 | Included | 1. age  2. sex  3. familial coronary disease  4. diabetes  5. hypertension  6. dyslipidaemia  7. current smoking  8. BMI | Increased relative risk of heart failure in IBD: aHR=2.03 (1.36-3.03). Risk increased among UC patients: aHR=2.06 (1.18-3.6) but not among CD patients. Increased risk of heart failure for systemic corticosteroid users: aHR=2.51 (1.93-4.57) but not for non-users. |
| Kristensen^3^,  2014, Denmark,  Nationwide,  1997-2011,  Mean: 6.4 years | IBD: 23,681  CD: 6,322 (26.7%)  UC: 17,359 (73.3%) | Mean 38.3 (SD: 18.3)  Only adult included | 53.7 | IBD: 533 | IBD: 36.8 vs 26.9 | Included | 1. age  2. sex  3. calendar year  4. income  5. cardiovascular pharmacotherapy (5 agents for hypertension, cholesterol-lowering, glucose lowering, antiplatelets, and loop diuretics)  6. comorbidity (7 diseases including hypertension, diabetes, atrial fibrillation, thromboembolism, vascular disease, COPD, and renal disease) | Patients with IBD have an increased risk of hospitalization for HF. IRR=1.37  (95% CI: 1.26-1.49),  CD: IRR=1.57  (1.31-1.89)  UC: IRR=1.33  (1.21-1.46)  especially during flares IBD: IRR=2.54  (2.13-3.04),  persistent activity  IBD: IRR=2.74  (2.25-3.33), but not for those in remission. |

aHR: adjusted hazard ratio; CD: Crohn’s disease; CI: confidence interval; CKD: chronic kidney diseases; COPD: chronic obstructive pulmonary disease; IBD(-U): inflammatory bowel disease (unclassified); IRR: incidence rate ratio; IQR: interquartile range; SD: standard deviation; UC: ulcerative colitis.

| **Table S2** ICD codes and SNOMED codes defining IBD. | | | | | |
| --- | --- | --- | --- | --- | --- |
| **IBD subtypes** ^a^ | **ICD-7  (1964-1968)** | **ICD-8  (1969-1986)** | **ICD-9  (1987-1996)** | **ICD-10  (1997-)** | **SNOMED codes ^b^** |
| UC | 572,20; 572,21; 578,03 | 563,1; 563,10; 569,02; 569,04 | 556 | K51 | D6255 or M41; M42; M43; M44; M463; or M47 |
| CD | 572,00; 572,09 | 563,00 | 555 | K50 | D6216 or M41; M42; M43; M44; M463; or M47 |
| IBD-U | UC + CD | UC + CD or 563; 563,0; 563,9; 563,98; 563,99 | UC + CD | UC + CD or K52.3 | D6214 or M41; M42; M43; M44; M463; or M47 |
| CD: Crohn’s disease; IBD(-U): inflammatory bowel disease (unclassified); ICD: International Classification of Disease; SNOMED: Systematized Nomenclature of Medicine; UC: ulcerative colitis.  ^a^ Diagnosis criteria^4^: ≥1 ICD code for IBD **AND** ≥1 SNOMED code for IBD (positive predictive value: 95%^5^)  ^b^ In SNOMED codes, D codes are the diagnostic codes, D6255 for example was the diagnostic code for UC. Meanwhile, codes starting with “M” (one unspecific SNOMED code) refer to all codes starting with the respective code.  Subtypes of IBD were defined according to the first ICD and SNOMED codes only (i.e., no information after start of follow-up contributed to the subtype definition); in case one individual had one ICD code for IBD and one unspecific SNOMED code (i.e., the "M" code), the IBD subtype was solely determined by the ICD code. | | | | | |

***Table S3*** *ICD codes for each phenotype of CD, UC, and IBD-U*

| **Montreal classification** | **Diagnostic codes** |
| --- | --- |
| CD location ^a^ |  |
| Ileal (L1) | K50.0 |
| Colonic (L2) | K50.1 |
| Ileocolonic or location not defined (L3/LX) | K50.8; K50.9 |
| Perianal disease modifier | Any of the diagnostic codes: K60.3; K60.4; K60.5; K61.0; K61.1; K61.2; K61.3;K61.4; K62.4 OR any of the surgical procedure codes: JHD20; JHD30; JHD33; JHD50; JHD60; JHD63; JHA00; JHA20; JHW96 |
| UC extent ^a^ |  |
| Proctitis (E1) | K51.2 |
| Left-sided colitis (E2) | K51.3; K51.5 |
| Extensive colitis (E3) | K51.0 |
| Extent not defined (EX) | K51.4; K51.8; K51.9 |
| Primary sclerosing cholangitis ^b^ | ICD-9 (1987-1996): 576B |
|  | ICD-10 (1997-): K830 |
| Other extraintestinal manifestations | ICD-9: 695C; 364; 713B; 720A; 720C; 720W; 720X |
|  | ICD-10: L52; L88; L98.2; H20; M07.4; M07.5; M07.6; |
|  | M09.1; M09.2; M45; M460; M461; M468; M469; M139; M255 |

CD: Crohn’s disease; E: extent; IBD(-U): inflammatory bowel disease (unclassified); ICD: International Classification of Disease; L: location; UC: ulcerative colitis.

^a^ The Montreal classification to validate and to define disease phenotypes was available since the use of ICD-10 in Sweden (1997-). All codes are captured in the Swedish National Patient Register (prospectively recorded in routine clinical practice).

In this study, we categorized Crohn's disease location into two groups: ileal (L1)/ileocolonic (L3)/unknown (LX) or colonic (L2), and categorized ulcerative colitis extent into three groups: proctitis (E1)/left-sided colitis (E2), extensive colitis (E3) or extent not defined (EX).

^b^ We restricted our use to ICD-9 and ICD-10 codes since we believe that earlier ICD codes for extraintestinal inflammation are less reliable, particularly for primary sclerosing cholangitis. The validity of the PSC codes has not formally been tested in Sweden^6^.

**Table S4a** Definitions of the exclusion criteria, outcomes, comorbidities, and medications

| **Exclusion** | **ICD codes** |
| --- | --- |
| Congenital heart disease | ICD-8: 746; 747.19-747,39; |
|  | ICD-9: 745-747; |
|  | ICD-10: Q20–Q26; |
| Heart transplant | ICD-9: V42B; |
|  | ICD-10: Z941, Swedish surgery/procedure codes: FQA; FQB; 3085; |
| **Outcome** | **ICD codes** |
| Heart failure | ICD-8: 427,00; 427,10; 428,99; |
|  | ICD-9: 428; |
|  | ICD-10: I50; I110; |
| **Comorbidities** | **ICD codes** |
| Any CVD ^a^ | ICD-8: 391-458 |
|  | ICD-9: 391-459 |
|  | ICD-10: I00-I99 |
| Ischemic heart disease | ICD-8: 410-411 (myocardial infarction); 412-414 (myocardial infarction: 412,01; 412,91) |
|  | ICD-9: 410 (myocardial infarction); 411-414 |
|  | ICD-10: I20; I21-I22 (myocardial infarction); I23-I25 |
| Arrhythmias | ICD-8: 427,20; 427,27; 427,28; 427,29; 427,9; 427,92 (atrial fibrillation/flutter); 795,99 |
|  | ICD-9: 426A; 426B; 426G; 426H; 426X; 427A; 427B; 427D (atrial fibrillation/flutter); 427E; 427F; 798B; 798C; |
|  | ICD-10: I441; I442; I452; I453; I456; I459; I460; I461; I469; I47; I48 (atrial fibrillation/flutter); I490; I495; R690; |
| Hypertension | ICD-8: 400-404 |
|  | ICD-9: 401-405 |
|  | ICD-10: I10; I119; I12-I15 |
|  | Medication ATC codes: see antihypertension medications below |
| Stroke | ICD-8: 430; 431; 433; 434; 436 |
|  | ICD-9: 430; 431; 434; 436 |
|  | ICD-10: I60; I61; I63; I64 |
| Anaemia | ICD-8: 280,08; 280,09; 281,10; 281,20; 281,30; 281,40; 281,98; 281,99; |
|  | ICD-9: 280X; 281B; 281C; 281E; 281W; 281X; |
|  | ICD-10: D50-D53; |
| Dyslipidemia | ICD-8: 279 |
|  | ICD-9: 272 |
|  | ICD-10: E78 ATC: C10 |
| Diabetes | ICD-8: 250 |
|  | ICD-9: 250 |
|  | ICD-10: E10-E14; O24 |
|  | Medication ATC codes: see antidiabetic medications below |
| Obesity | ICD-8: 277 |
|  | ICD-9: 278A; 278B |
|  | ICD-10: E65-E66 |
| Sleep problems | ICD-8: 347; 780,60 |
|  | ICD-9: 347; 780F |
|  | ICD-10: F51; G470- G474; G478; G479 |
| Chronic kidney diseases | ICD-8: 585; 586; Y29,01 |
|  | ICD-9: 585; 586; 753B; V42A; V45B; V56 |
|  | ICD-10: N18; N19; N26; T824; Y841; Q61; Z49; Z992; Z940 |
| COPD (only if diagnosed ≥40 years) ^b^ | ICD-8: 491; 492; |
|  | ICD-9: 491; 492; 496; |
|  | ICD-10: J41- J44; |
| Autoimmune diseases | ICD-8: 135; 136,03; 136,07; 242,00; 242,09; 242,10; 242,2; 244; 245; 246; 255,1; 269,0; 287,0; 323,00; 340; 341,01; 354; 355; 390; 391; 392; 443,0; 443,1; 446,0; 446,1; 446,2; 446,30; 446,38; 446,9; 447; 540-543; 571,9; 693,99; 694,00; 694,02; 696; 709,05; 711; 712; 713,1; 714; 715; 716,0; 716,1; 717; 726; 733,00; 734,0; 734,1; 734,90; 734,91; 734,98; 734,99 |
|  | ICD-9: 099D; 135; 136B; 242A; 242B; 242C; 242D; 242X; 244; 245; 255E; 287A; 323G; 340; 341A; 357A; 358A; 390-392; 443A; 443B; 446A; 446B; 446C; 446E; 446E; 446F; 446H; 447G; 540-543; 571G; 579A; 694A; 694C; 694E; 694F; 694G; 696; 709A; 710A; 710B; 710D; 710E; 710I; 714; 720; 725 |
|  | ICD-10: D690; D86; E034; E035; E038; E039; E050; E051; E052; E055; E058; E059; E061; E063; E069; E271; E272; G040; G048; G35; G360; G610; G611; G618; G619; G700; I00; I010; I011; I012; I018; I019; I020; I029; I730; I731; K35-K37; K743; K900; L100; L12; L130; L40; L80; M02; M050; M051; M052; M053; M058; M059; M060; M061; M062; M063; M064; M068; M069; M080; M081; M082; M083; M084; M088; M089; M090; M091; M092; M098; M300-M303; M310; M313-M317; M32; M330-M332; M339; M34; M350-M353; M45 |

| **Co-medications** | **ATC codes** |
| --- | --- |

| Aspirin | B01AC06 |
| --- | --- |
| Non-aspirin anti-platelet medications | B01AC excluding aspirin (B01AC06) |
| Statins | C10AA |
| Non-statin lipid-lowering medications | C10AB, C10AC, C10AD, C10AX01-14 |
| Anticoagulants | B01AA, B01AE, B01AF, B01AX |
| Antidiabetics | A10 |
| Antihypertensives | C02, C03AA-AB, C03BA, C03CA, C03DA, C03EA, C08CA, C08DA, C08DB, C09A, C09BA, C09BB, C09CA, C09DA, C09DB01 |

ATC: anatomical therapeutic chemical; CVD: cardiovascular disease; COPD: chronic obstructive pulmonary disease; ICD: International Classification of Disease;

^a^ Only in subgroup analysis.

^b^ As in our earlier research^7^, for COPD to be a proxy for heavy smoking, we restricted patients diagnosed after 40 years of age to avoid misclassification due to genetic susceptibility for COPD.

| **Table S4b** IBD-related surgical procedure codes | | |
| --- | --- | --- |
| Classification of surgical procedures | 5/6th edition (1963-1996) | 7th edition (1997-) |
| Colectomy |  |  |
| 1) Subtotal colectomy with end ileostomy |  |  |
| Colectomy and ileostomy with closure of the rectum | 4651 | JFH10 |
| Laparoscopic colectomy and ileostomy |  | JFH11 |
| Other colectomy |  | JFH96 |
| 2) Colectomy with ileorectal anastomosis |  |  |
| Colectomy with ileorectal anastomosis | 4650 | JFH00 |
| Laparoscopic colectomy with ileorectal anastomosis |  | JFH01 |
| Ileorectal anastomosis |  | JFC40 |
| Laparoscopic ileorectal anastomosis |  | JFC41 |
| Closure of enterostomy with anastomosis to the rectum |  | JFG29 |
| Closure of enterostomy with anastomosis to the colon |  | JFG26 |
| 3) Partial colectomies |  |  |
| Right-sided colectomy | 4641 | JFB30, JFB31 |
| Resection of the colon transversum | 4643 | JGB40, JFB41 |
| Left-sided colectomy | 4640 | JFB43, JFB44 |
| Resection of the sigmoid colon | 4644 | JFB46, JFB47 |
| Other colon resection | 4649 | JFB50, JFB51 |
| 4) Proctocolectomy with ileal pouch-anal anastomosis |  |  |
| Colectomy, rectal mucosectomy and ileoanal anastomosis *without* ileostomy. |  | JFH30 |
| Colectomy, rectal mucosectomy and ileoanal anastomosis *and* ileostomy. |  | JFH33 |
| Mucosectomy and ileoanal anastomosis after previous colectomy. | 4654 | JGB50 |
| Extirpation of rectum or making of an ileoanal anastomosis after previous colectomy. |  | JGB60 |
| 5) Continent ileostomy at time of colectomy |  |  |
| Proctocolectomy with continent ileostomy, “Kock” | 4653 | JFH40 |
| Converting a conventional ileostomy to a continent ileostomy |  | JFG60 |
| 6) Proctocolectomy |  |  |
| Proctocolectomy with ileostomy | 4652 | JFH20 |
| Other bowel surgery |  |  |
| Strictureplasty to the small bowel |  | JFA60 |
| Strictureplasty to the colon |  | JFA63 |
| Closure of small intestinal fistula |  | JFA76 |
| Closure of colonic fistula |  | JFA86 |
| Colonic and/or small bowel resection | 4630, 4631, 4640-4649 | JFB |
| Formation of stoma |  | JFF |
| Operations on intestinal stoma or reservoir |  | JFG |
| Other operation of the small bowel and/or colon | 4660-4668, 4700-4739, 4790-4798 | JFW96 |
| Other laparoscopic operation of the small bowel and/or colon |  | JFW97 |
| Rectal resection | 4820-4828 | JGB |
| Perianal surgery |  |  |
| Perianal incision and drainage | 4900 | JHA00 |
| Dilatation of the anal sphincter | 4960 | JHD00 |
| Lay open or excision of perianal fistula | 4920, 4922-4924 | JHD20 |
| Partial lay open or excision of perianal fistula (including seton placement) | 4970-4971 | JHD30 |
| Completion lay open or excision of perianal fistula |  | JHD33 |
| Excision of perianal fistula with advancement flap |  | JHD50 |
| Occlusion of perianal fistula with collagen plug |  | JHD60 |
| Occlusion of perianal fistula with fibrin glue |  | JHD63 |
| Other anal or perianal surgery (e.g., examination under anesthesia) | 4999 | JHW96 |
| IBD: Inflammatory bowel disease. |  |  |

| **Table S4c** IBD medications: biologics and steroids | |
| --- | --- |
| Medications | ATC code |
| Steroids |  |
| Systemic corticosteroids |  |
| Betamethasone | H02AB01 |
| Dexamethasone | H02AB02 |
| Methylprednisolone | H02AB04 |
| Prednisolone | H02AB06 |
| Prednisone | H02AB07 |
| Hydrocortisone | H02AB09 |
| Cortisone | H02AB10 |
| Corticosteroids acting locally |  |
| Hydrocortisone | A07EA02 |
| Budesonide | A07EA06 |
| Biologics |  |
| Anti-TNF treatment |  |
| Infliximab | L04AB02 (L04AA12 before 2008) |
| Adalimumab | L04AB04 (L04AA17 before 2008) |
| Golimumab | L04AB06 |
| Other biologics/tofacitinib |  |
| Vedolizumab | L04AA33 |
| Tofacitinib | L04AA29 |
| Ustekinumab | L04AC05 |
| ATC: Anatomical Therapeutic Chemical; IBD: inflammatory bowel disease; TNF: tumor necrosis factor. | |

| **Table S5** Clinical phenotypes of patients with IBD, n (%) | | | |
| --- | --- | --- | --- |
|  | Subtypes of IBD | | |
|  | CD | UC | IBD-U |
| Montreal Classification of CD at index date |  |  |  |
| No location information | 6140 (25.3) | - | - |
| L1, L3/LX (Ileal, ileocolonic or location not defined) | 14613 (60.1) | - | - |
| L2 (Colonic) | 3550 (14.6) | - | - |
| B1 (Non-stricturing, non-penetrating) | 22727 (93.5) | - | - |
| B2/B3 (Stricturing or penetrating) | 1566 (6.4) | - | - |
| Perianal | 1280 (5.3) | - | - |
| Montreal Classification of UC at index date |  |  |  |
| No extent information | - | 10689 (23.4) | - |
| E1/E2 (Proctitis, left-sided colitis) | - | 13052 (28.6) | - |
| E3 (Extensive colitis) | - | 7143 (15.6) | - |
| EX (Extent not defined) | - | 14825 (32.4) | - |
| Extraintestinal manifestations at index date |  |  |  |
| Primary sclerosing cholangitis | 151 (0.6) | 725 (1.6) | 183 (1.6) |
| Other extraintestinal manifestations | 1418 (5.8) | 1705 (3.7) | 791 (6.7) |
| CD: Crohn’s disease; E: Extent; IBD(-U): inflammatory bowel disease (unclassified); L: location; UC: ulcerative colitis. | | | |

| **Table S6** Subgroup analyses of heart failure in patients with IBD and their matched reference individuals | | | | |
| --- | --- | --- | --- | --- |
| Group | Heart failure | | | |
|  | No. of events (IR, per 10,000 person-years) | | IR difference (95%CI), per 10,000 person-years | HR (95%CI) ^a^ |
|  | Patients | References |  |  |
| Sex |  |  |  |  |
| Male | 3115 (53.9) | 11835 (41.8) | 12.1 (10.0 to 14.1) | 1.16 (1.11 to 1.21) |
| Female | 2467 (46.5) | 8508 (33.6) | 12.8 (10.9 to 14.8) | 1.23 (1.16 to 1.30) |
| Age at index date, years |  |  |  |  |
| <18 | 20 (1.9) | 32 (0.6) | 1.3 (0.4 to 2.2) | 2.72 (1.45 to 5.11) |
| 18-<40 | 321 (6.4) | 1056 (4.4) | 2.1 (1.3 to 2.8) | 1.34 (1.17 to 1.53) |
| 40-<60 | 1470 (41.0) | 5811 (33.6) | 7.4 (5.1 to 9.6) | 1.09 (1.02 to 1.16) |
| ≥60 | 3771 (252.3) | 13444 (193.0) | 59.4 (50.7 to 68.1) | 1.22 (1.16 to 1.27) |
| Calendar period at index date |  |  |  |  |
| 1969-1989 | 738 (49.8) | 3302 (44.7) | 5.0 (1.1 to 8.9) | 1.11 (1.01 to 1.22) |
| 1990-2001 | 2501 (52.6) | 9670 (41.6) | 11.0 (8.7 to 13.2) | 1.21 (1.15 to 1.27) |
| 2002-2009 | 1636 (49.0) | 5496 (34.6) | 14.4 (11.8 to 16.9) | 1.16 (1.08 to 1.23) |
| 2010-2017 | 707 (46.7) | 1875 (26.4) | 20.3 (16.7 to 23.9) | 1.26 (1.13 to 1.40) |
| Educational attainment, years |  |  |  |  |
| 0-9 | 2207 (98.2) | 8191 (75.9) | 22.3 (17.9 to 26.7) | 1.14 (1.06 to 1.22) |
| 10-12 | 1727 (40.3) | 5914 (29.9) | 10.5 (8.4 to 12.5) | 1.21 (1.12 to 1.31) |
| ≥13 | 685 (28.3) | 2339 (19.3) | 9.0 (6.8 to 11.3) | 1.28 (1.09 to 1.50) |
| Missing | 963 (44.9) | 3899 (35.8) | 9.1 (6.1 to 12.2) | 1.17 (1.07 to 1.28) |
| Number of healthcare visits ^b^ |  |  |  |  |
| 0 | 3136 (42.3) | 14904 (34.0) | 8.3 (6.7 to 9.9) | 1.22 (1.17 to 1.28) |
| 1 | 950 (55.4) | 2546 (47.3) | 8.1 (4.1 to 12.0) | 1.10 (0.93 to 1.31) |
| 2-3 | 781 (69.0) | 1699 (59.8) | 9.1 (3.5 to 14.7) | 1.08 (0.87 to 1.34) |
| ≥4 | 715 (86.6) | 1194 (77.5) | 9.1 (1.4 to 16.8) | 1.27 (1.01 to 1.61) |
| CI: confidence interval; HR: hazard ratio; IBD: inflammatory bowel disease; IR: incidence rate. | | | | |
| ^a^ Conditioned on the matching variables (birth year, sex, county of residence, and calendar year) and further adjusted for country of birth, educational attainment, number of healthcare visits, ischemic heart disease, arrhythmias, hypertension, anemia, dyslipidemia, diabetes, obesity, sleep problems, chronic kidney diseases, chronic obstructive pulmonary disease (only if diagnosed ≥ 40 years), and autoimmune diseases.  ^b^ Defined as the number of healthcare visits between 2 years and 6 months before the index date. | | | | |

***Table S7*** *Subgroup analyses of heart failure in patients with CD, UC, IBD-U and their matched reference individuals*

| Group | CD | | | |  | UC | | | | |  | IBD-U | | | |
| --- | --- | --- | --- | --- | --- | --- | --- | --- | --- | --- | --- | --- | --- | --- | --- |
|  | No. of events  (IR, per 10,000 person-years) | | IR difference (95%CI), per 10,000  person-years | HR (95%CI) ^a^ |  | No. of events  (IR, per 10,000 person-years) | | IR difference (95%CI), per 10,000 person-years | HR (95%CI) ^a^ | |  | No. of events (IR, per 10,000 person-years) | | IR difference (95%CI), per 10,000  person-years | HR (95%CI) ^a^ |
|  | Patients | References |  |  |  | Patients | References |  |  |  |  | Patients | References |  |  |
| Sex |  |  |  |  |  |  |  |  | |  |  |  |  |  |  |
| Male | 808 (48.9) | 3133 (38.3) | 10.6 (7.0 to 14.2) | 1.17 (1.07 to 1.29) |  | 1906 (54.7) | 7385 (43.5) | 11.2 (8.5 to 13.8) | 1.12 (1.06 to 1.19) | |  | 401 (62.1) | 1317 (41.6) | 20.5 (14.1 to 27.0) | 1.33 (1.16 to 1.52) |
| Female | 806 (45.1) | 2667 (30.8) | 14.2 (10.9 to 17.6) | 1.39 (1.26 to 1.53) |  | 1294 (44.5) | 4771 (34.9) | 9.7 (7.1 to 12.3) | 1.15 (1.07 to 1.24) | |  | 367 (59.6) | 1070 (36.3) | 23.3 (16.8 to 29.8) | 1.22 (1.06 to 1.42) |
| Age at index date, years |  |  |  |  |  |  |  |  |  | |  |  |  |  |  |
| <18 | 5 (1.2) | 15 (0.7) | 0.5 (-0.6 to 1.6) | 1.10 (0.31 to 3.89) |  | 13 (2.7) | 15 (0.6) | 2.1 (0.6 to 3.6) | 4.21 (1.83 to 9.67) | |  | 2 (1.4) | 2 (0.3) | 1.2 (-0.9 to 3.2) | 3.96 (0.24 to 64.55) |
| 18-<40 | 126 (7.7) | 338 (4.2) | 3.4 (2.0 to 4.9) | 1.65 (1.31 to 2.07) |  | 162 (5.7) | 626 (4.5) | 1.2 (0.2 to 2.1) | 1.18 (0.98 to 1.43) | |  | 33 (6.6) | 92 (3.8) | 2.8 (0.4 to 5.2) | 1.66 (1.06 to 2.61) |
| 40-<60 | 470 (47.0) | 1832 (37.3) | 9.7 (5.1 to 14.3) | 1.17 (1.04 to 1.31) |  | 824 (37.8) | 3394 (32.7) | 5.1 (2.3 to 7.9) | 1.01 (0.93 to 1.10) | |  | 176 (42.8) | 585 (29.1) | 13.7 (7.0 to 20.5) | 1.30 (1.07 to 1.57) |
| ≥60 | 1013 (263.7) | 3615 (193.1) | 70.6 (53.2 to 88.0) | 1.29 (1.19 to 1.41) |  | 2201 (245.3) | 8121 (198.3) | 47.1 (36.0 to 58.2) | 1.18 (1.12 to 1.25) | |  | 557 (261.3) | 1708 (171.1) | 90.2 (67.1 to 113.4) | 1.25 (1.11 to 1.41) |
| Calendar period at index date |  |  |  |  |  |  |  |  |  | |  |  |  |  |  |
| 1969-1989 | 249 (42.4) | 1165 (39.5) | 2.8 (-2.9 to 8.6) | 1.15 (0.98 to 1.35) |  | 438 (54.2) | 1963 (49.0) | 5.2 (-0.3 to 10.7) | 1.04 (0.92 to 1.18) | |  | 51 (58.7) | 174 (40.6) | 18.0 (0.8 to 35.2) | 1.70 (1.14 to 2.54) |
| 1990-2001 | 718 (48.8) | 2722 (37.4) | 11.4 (7.5 to 15.2) | 1.31 (1.19 to 1.45) |  | 1523 (53.1) | 6039 (43.5) | 9.6 (6.7 to 12.5) | 1.14 (1.07 to 1.22) | |  | 260 (62.3) | 909 (43.7) | 18.6 (10.5 to 26.7) | 1.39 (1.17 to 1.64) |
| 2002-2009 | 474 (49.9) | 1441 (31.4) | 18.4 (13.7 to 23.2) | 1.32 (1.16 to 1.50) |  | 902 (46.6) | 3204 (35.1) | 11.5 (8.2 to 14.7) | 1.11 (1.02 to 1.21) | |  | 260 (57.4) | 851 (39.2) | 18.2 (10.7 to 25.6) | 1.10 (0.93 to 1.30) |
| 2010-2017 | 173 (39.9) | 472 (23.1) | 16.8 (10.5 to 23.1) | 1.20 (0.97 to 1.49) |  | 337 (43.4) | 950 (26.2) | 17.2 (12.3 to 22.1) | 1.28 (1.11 to 1.48) | |  | 197 (64.9) | 453 (31.6) | 33.3 (23.8 to 42.8) | 1.32 (1.06 to 1.63) |
| Educational attainment, years |  |  |  |  |  |  |  |  |  | |  |  |  |  |  |
| 0-9 | 612 (86.6) | 2239 (68.5) | 18.2 (10.7 to 25.6) | 1.23 (1.07 to 1.41) |  | 1294 (102.3) | 4941 (79.6) | 22.8 (16.8 to 28.8) | 1.11 (1.02 to 1.21) | |  | 301 (108.9) | 1011 (77.1) | 31.7 (18.5 to 44.9) | 1.11 (0.91 to 1.36) |
| 10-12 | 505 (39.9) | 1619 (27.9) | 12.0 (8.2 to 15.7) | 1.29 (1.10 to 1.51) |  | 975 (38.7) | 3504 (30.0) | 8.7 (6.1 to 11.4) | 1.15 (1.04 to 1.28) | |  | 247 (49.2) | 791 (34.0) | 15.2 (8.7 to 21.8) | 1.35 (1.08 to 1.69) |
| ≥13 | 184 (29.3) | 634 (18.1) | 11.2 (6.7 to 15.7) | 1.55 (1.13 to 2.14) |  | 373 (25.0) | 1372 (19.4) | 5.6 (2.8 to 8.3) | 1.05 (0.84 to 1.31) | |  | 128 (42.9) | 333 (21.5) | 21.4 (13.6 to 29.1) | 1.97 (1.31 to 2.97) |
| Missing | 313 (37.2) | 1308 (30.6) | 6.5 (2.1 to 11.0) | 1.18 (1.01 to 1.38) |  | 558 (49.9) | 2339 (41.0) | 8.9 (4.4 to 13.3) | 1.11 (0.99 to 1.25) | |  | 92 (49.9) | 252 (27.1) | 22.8 (12.1 to 33.5) | 1.75 (1.22 to 2.52) |
| Number of healthcare visits ^b^ |  |  |  |  |  |  |  |  |  | |  |  |  |  |  |
| 0 | 884 (39.2) | 4276 (30.8) | 8.4 (5.7 to 11.2) | 1.36 (1.24 to 1.49) |  | 1908 (43.0) | 9064 (35.9) | 7.1 (5.0 to 9.1) | 1.14 (1.08 to 1.22) | |  | 344 (47.5) | 1564 (33.2) | 14.3 (9.0 to 19.6) | 1.39 (1.19 to 1.61) |
| 1 | 274 (51.7) | 741 (44.8) | 6.9 (-0.0 to 13.8) | 0.94 (0.66 to 1.34) |  | 531 (54.4) | 1476 (48.8) | 5.7 (0.4 to 10.9) | 1.07 (0.86 to 1.34) | |  | 145 (69.0) | 329 (46.9) | 22.1 (9.8 to 34.4) | 1.55 (0.96 to 2.49) |
| 2-3 | 238 (65.1) | 467 (56.2) | 9.0 (-0.7 to 18.7) | 1.18 (0.77 to 1.81) |  | 398 (67.0) | 977 (62.1) | 4.9 (-2.7 to 12.5) | 0.99 (0.73 to 1.32) | |  | 145 (83.8) | 255 (58.8) | 25.0 (9.6 to 40.5) | 1.32 (0.73 to 2.40) |
| ≥4 | 218 (75.1) | 316 (69.8) | 5.3 (-7.3 to 17.8) | 1.16 (0.71 to 1.88) |  | 363 (95.1) | 639 (78.0) | 17.1 (5.6 to 28.6) | 1.74 (1.24 to 2.46) | |  | 134 (87.0) | 239 (88.8) | -1.7 (-20.3 to 16.8) | 0.94 (0.56 to 1.58) |
| CD: Crohn's disease; CI: confidence interval; HR: hazard ratio; IBD(-U): inflammatory bowel disease (unclassified); IR: incidence rate; NA: not available; UC: ulcerative colitis. | | | | | | | | | | | | | | | |
| ^a^ Conditioned on the matching variables (birth year, sex, county of residence, and calendar year) and further adjusted for country of birth, educational attainment, number of healthcare visits, ischemic heart disease, arrhythmias, hypertension, anemia, dyslipidemia, diabetes, obesity, sleep problems, chronic kidney diseases, chronic obstructive pulmonary disease (only if diagnosed ≥ 40 years), and autoimmune diseases.  ^b^ Defined as the number of healthcare visits between 2 years and 6 months before the index date. | | | | | | | | | | | | | | | |

| | **Table S8** Incident heart failure in patients with IBD and their matched reference individuals, stratified by the phenotypes of the Montreal Classification | | | | | | --- | --- | --- | --- | --- | | Group | Heart failure | | | | | No. of events (IR, per 10,000 person-years) | | IR difference (95%CI), per 10,000 person-years | HR (95%CI) ^a^ | | Patients | References | | CD |  |  |  |  | | No location information/ICD code before 1997 | 674 (46.9) | 2829 (39.5) | 7.4 (3.6 to 11.2) | 1.25 (1.13 to 1.38) | | L1, L3/LX (Ileal, ileocolonic or location not defined) | 738 (46.4) | 2327 (30.3) | 16.1 (12.5 to 19.7) | 1.29 (1.16 to 1.42) | | L2 (Colonic) | 202 (48.5) | 644 (31.9) | 16.6 (9.4 to 23.7) | 1.34 (1.11 to 1.62) | | Perianal | 35 (26.1) | 101 (15.4) | 10.7 (1.5 to 19.9) | 1.29 (0.75 to 2.22) | | Primary sclerosing cholangitis | 7 (43.1) | 38 (44.2) | -1.1 (-36.0 to 33.8) | 1.37 (0.44 to 4.24) | | Other extraintestinal manifestations | 80 (60.3) | 177 (27.6) | 32.7 (18.9 to 46.5) | 1.35 (0.90 to 2.03) | | UC |  |  |  |  | | No extent information/ICD code before 1997 | 1311 (56.3) | 5388 (47.0) | 9.3 (6.0 to 12.6) | 1.14 (1.06 to 1.23) | | E1/E2 (Proctitis, left-sided colitis) | 598 (42.2) | 2228 (33.8) | 8.4 (4.8 to 12.1) | 1.06 (0.96 to 1.18) | | E3 (Extensive colitis) | 336 (38.9) | 1250 (30.3) | 8.6 (4.1 to 13.1) | 1.08 (0.94 to 1.25) | | EX (Extent not defined) | 955 (53.6) | 3290 (38.8) | 14.7 (11.1 to 18.4) | 1.18 (1.08 to 1.28) | | Primary sclerosing cholangitis | 35 (37.1) | 150 (29.6) | 7.5 (-5.6 to 20.7) | 1.03 (0.64 to 1.66) | | Other extraintestinal manifestations | 117 (71.3) | 275 (35.2) | 36.1 (22.5 to 49.7) | 1.58 (1.17 to 2.13) | | IBD-U |  |  |  |  | | Primary sclerosing cholangitis | 8 (43.0) | 29 (28.0) | 15.0 (-16.5 to 46.4) | 1.03 (0.28 to 3.83) | | Other extraintestinal manifestations | 42 (66.6) | 110 (35.7) | 30.9 (9.7 to 52.1) | 1.23 (0.71 to 2.11) | | CD: Crohn’s disease; CI: confidence interval; E: Extent; HR: hazard ratio; IBD(-U): inflammatory bowel disease (unclassified); ICD: ICD: International Classification of Disease; IR: incident rate; L: location; UC: ulcerative colitis. | | | | | | ^a^ Conditioned on the matching variables (birth year, sex, county of residence, and calendar year) and further adjusted for country of birth, educational attainment, number of healthcare visits, ischemic heart disease, arrhythmias, hypertension, anemia, dyslipidemia, diabetes, obesity, sleep problems, chronic kidney diseases, chronic obstructive pulmonary disease (only if diagnosed ≥ 40 years), and autoimmune diseases. | | | | | |
| --- | --- | --- | --- | --- | --- | --- | --- | --- | --- | --- | --- | --- | --- | --- | --- | --- | --- | --- | --- | --- | --- | --- | --- | --- | --- | --- | --- | --- | --- | --- | --- | --- | --- | --- | --- | --- | --- | --- | --- | --- | --- | --- | --- | --- | --- | --- | --- | --- | --- | --- | --- | --- | --- | --- | --- | --- | --- | --- | --- | --- | --- | --- | --- | --- | --- | --- | --- | --- | --- | --- | --- | --- | --- | --- | --- | --- | --- | --- | --- | --- | --- | --- | --- | --- | --- | --- | --- | --- | --- | --- | --- | --- | --- | --- | --- | --- | --- | --- | --- | --- | --- | --- | --- | --- | --- | --- | --- | --- | --- | --- | --- |

| **Table S9** Sensitivity analyses of incident heart failure in patients with IBD and their matched reference individuals | | | | |
| --- | --- | --- | --- | --- |
| Group | Heart failure | | | |
|  | No. of events (IR, per 10,000 person-years) | | IR difference (95%CI), per 10,000 person-years | HR (95%CI) |
|  | Patients | References |  |  |
| Individuals with available educational attainment ^a^ |  |  |  |  |
| Overall IBD | 4619 (51.6) | 16444 (38.5) | 13.1 (11.5 to 14.7) | 1.18 (1.13 to 1.22) |
| CD | 1301 (50.0) | 4492 (35.7) | 14.3 (11.4 to 17.2) | 1.28 (1.19 to 1.38) |
| UC | 2642 (50.1) | 9817 (39.3) | 10.8 (8.7 to 12.8) | 1.13 (1.07 to 1.18) |
| IBD-U | 676 (62.8) | 2135 (41.2) | 21.6 (16.6 to 26.6) | 1.23 (1.11 to 1.37) |
| Individuals with an index date of January 2006 or later ^b^ |  |  |  |  |
| Overall IBD | 1424 (47.9) | 4209 (30.1) | 17.9 (15.2 to 20.5) | 1.18 (1.10 to 1.27) |
| CD | 375 (44.5) | 1058 (26.4) | 18.0 (13.3 to 22.8) | 1.28 (1.10 to 1.48) |
| UC | 707 (44.6) | 2253 (30.3) | 14.2 (10.7 to 17.8) | 1.14 (1.03 to 1.26) |
| IBD-U | 342 (63.1) | 898 (34.9) | 28.1 (21.1 to 35.2) | 1.22 (1.04 to 1.43) |
| Discard the first year of follow-up from analysis ^a^ |  |  |  |  |
| Overall IBD | 5041 (49.0) | 18188 (37.0) | 12.0 (10.6 to 13.5) | 1.14 (1.10 to 1.19) |
| CD | 1471 (45.9) | 5195 (33.5) | 12.4 (9.9 to 15.0) | 1.24 (1.15 to 1.33) |
| UC | 2907 (49.0) | 10947 (38.8) | 10.1 (8.2 to 12.1) | 1.09 (1.04 to 1.14) |
| IBD-U | 663 (57.8) | 2046 (37.3) | 20.5 (15.8 to 25.2) | 1.22 (1.10 to 1.36) |
| Discard the first three years of follow-up from analysis ^a^ |  |  |  |  |
| Overall IBD | 4338 (49.7) | 15331 (37.1) | 12.6 (11.0 to 14.2) | 1.13 (1.09 to 1.18) |
| CD | 1274 (46.5) | 4413 (33.6) | 12.9 (10.2 to 15.7) | 1.22 (1.13 to 1.31) |
| UC | 2533 (50.1) | 9300 (39.1) | 10.9 (8.8 to 13.0) | 1.08 (1.02 to 1.13) |
| IBD-U | 531 (57.2) | 1618 (36.9) | 20.3 (15.2 to 25.5) | 1.22 (1.08 to 1.37) |
| Heart failure defined as ≥2 diagnoses ^a^ |  |  |  |  |
| Overall IBD | 3674 (32.9) | 13603 (25.2) | 7.7 (6.6 to 8.8) | 1.18 (1.13 to 1.23) |
| CD | 1074 (31.0) | 3819 (22.5) | 8.5 (6.5 to 10.5) | 1.31 (1.21 to 1.42) |
| UC | 2086 (32.5) | 8193 (26.6) | 5.9 (4.4 to 7.4) | 1.11 (1.05 to 1.17) |
| IBD-U | 514 (40.4) | 1591 (25.8) | 14.6 (10.9 to 18.3) | 1.29 (1.15 to 1.46) |
| Additionally censored at date of first IBD-related surgery ^a^ |  |  |  |  |
| Overall IBD | 4520 (51.3) | 19355 (37.1) | 14.2 (12.7 to 15.8) | 1.19 (1.15 to 1.24) |
| CD | 1204 (48.5) | 5427 (33.5) | 15.0 (12.1 to 17.9) | 1.26 (1.17 to 1.36) |
| UC | 2652 (50.3) | 11642 (38.8) | 11.5 (9.5 to 13.6) | 1.15 (1.09 to 1.21) |
| IBD-U | 664 (62.9) | 2286 (38.1) | 24.9 (19.8 to 29.9) | 1.27 (1.14 to 1.42) |
| Additionally censored at date of first steroids prescription after IBD diagnosis ^a, c^ |  |  |  |  |
| Overall IBD | 435 (41.6) | 2910 (25.1) | 16.5 (12.5 to 20.5) | 1.19 (1.04 to 1.35) |
| CD | 108 (39.5) | 727 (22.0) | 17.5 (9.9 to 25.1) | 1.15 (0.89 to 1.49) |
| UC | 195 (34.2) | 1526 (24.8) | 9.4 (4.4 to 14.3) | 1.11 (0.92 to 1.33) |
| IBD-U | 132 (65.4) | 657 (30.6) | 34.7 (23.4 to 46.1) | 1.40 (1.08 to 1.80) |
| Additionally censored at date of first biological therapy after IBD diagnosis ^a, c^ |  |  |  |  |
| Overall IBD | 1379 (50.9) | 4209 (30.1) | 20.8 (18.0 to 23.6) | 1.19 (1.11 to 1.28) |
| CD | 355 (49.0) | 1058 (26.5) | 22.5 (17.2 to 27.8) | 1.24 (1.07 to 1.44) |
| UC | 690 (46.5) | 2253 (30.4) | 16.1 (12.4 to 19.8) | 1.16 (1.05 to 1.29) |
| IBD-U | 334 (66.9) | 898 (34.9) | 31.9 (24.4 to 39.5) | 1.23 (1.05 to 1.44) |
| CD: Crohn's disease; CI: confidence interval; HR: hazard ratio; IBD(-U): inflammatory bowel disease (unclassified); IR: incidence rate; UC: ulcerative colitis. | | | | |
| ^a^ Conditioned on the matching variables (birth year, sex, county of residence, and calendar year) and further adjusted for country of birth, educational attainment, number of healthcare visits, ischemic heart disease, arrhythmias, hypertension, anemia, dyslipidemia, diabetes, obesity, sleep problems, chronic kidney diseases, chronic obstructive pulmonary disease, and autoimmune diseases. | | | | |
| ^b^ Further adjusted for aspirin, non-aspirin anti-platelet medications, statins, non-statin lipid-lowering medications, anticoagulation medications, antidiabetic medications, and antihypertensive agents. | | | | |
| ^c^ Restricted the analysis to individuals with an index date of January 2006 or later. | | | | |

| **Table S10** Characteristics of patients with IBD and their IBD-free full siblings, n (%) | | | | | | | | |
| --- | --- | --- | --- | --- | --- | --- | --- | --- |
|  | Full siblings | Patients | | Subtypes of IBD | | | | |
|  |  |  |  | CD | | UC | | IBD-U |
| N | 95239 | | 52761 | | 15771 | | 29567 | 7423 |
| Age at index date, years ^a^ |  | |  | |  | |  |  |
| Mean ± SD | 38.9 ± 16.8 | | 37.8 ± 16.3 | | 35.7 ± 16.4 | | 38.5 ± 15.7 | 39.3 ± 17.9 |
| Median (IQR) | 38.3 (25.4-51.9) | | 36.1 (24.5-50.1) | | 33.2 (22.1-48.3) | | 37.0 (26.0-50.2) | 37.7 (24.4-53.7) |
| <18 | 9145 (9.6) | | 5711 (10.8) | | 2274 (14.4) | | 2517 (8.5) | 920 (12.4) |
| 18-<40 | 42180 (44.3) | | 24608 (46.6) | | 7489 (47.5) | | 14052 (47.5) | 3067 (41.3) |
| 40-<60 | 32542 (34.2) | | 16594 (31.5) | | 4517 (28.6) | | 9825 (33.2) | 2252 (30.3) |
| ≥60 | 11372 (11.9) | | 5848 (11.1) | | 1491 (9.5) | | 3173 (10.7) | 1184 (16.0) |
| Female | 46045 (48.4) | | 24816 (47.0) | | 7965 (50.5) | | 13310 (45.0) | 3541 (47.7) |
| Born in Nordic country ^b^ | 92012 (96.6) | | 51388 (97.4) | | 15229 (96.6) | | 28922 (97.8) | 7237 (97.5) |
| Calendar period at index date ^a^ |  | |  | |  | |  |  |
| 1969-1989 | 6020 (6.3) | | 3038 (5.8) | | 1213 (7.7) | | 1664 (5.6) | 161 (2.2) |
| 1990-2001 | 29065 (30.5) | | 15559 (29.5) | | 4781 (30.3) | | 9372 (31.7) | 1406 (18.9) |
| 2002-2009 | 31137 (32.7) | | 17375 (32.9) | | 4987 (31.6) | | 9992 (33.8) | 2396 (32.3) |
| 2010-2017 | 29017 (30.5) | | 16789 (31.8) | | 4790 (30.4) | | 8539 (28.9) | 3460 (46.6) |
| Educational attainment, years |  | |  | |  | |  |  |
| 0-9 | 19699 (20.7) | | 10089 (19.1) | | 3237 (20.5) | | 5386 (18.2) | 1466 (19.8) |
| 10-12 | 38583 (40.5) | | 22085 (41.9) | | 6350 (40.3) | | 12624 (42.7) | 3111 (41.9) |
| ≥13 | 21817 (22.9) | | 13152 (24.9) | | 3281 (20.8) | | 8009 (27.1) | 1862 (25.1) |
| Missing | 15140 (15.9) | | 7435 (14.1) | | 2903 (18.4) | | 3548 (12.0) | 984 (13.3) |
| Number of healthcare visits ^c^ |  | |  | |  | |  |  |
| 0 | 70865 (74.4) | | 31879 (60.4) | | 9169 (58.1) | | 18834 (63.7) | 3876 (52.2) |
| 1 | 11758 (12.4) | | 8618 (16.3) | | 2568 (16.3) | | 4773 (16.1) | 1277 (17.2) |
| 2-3 | 7709 (8.1) | | 6608 (12.5) | | 2058 (13.1) | | 3419 (11.6) | 1131 (15.2) |
| ≥4 | 4907 (5.2) | | 5656 (10.7) | | 1976 (12.5) | | 2541 (8.6) | 1139 (15.3) |
| Disease history before index date ^a^ | , d | |  | |  | |  |  |
| Any CVD | 9550 (10.0) | | 7202 (13.7) | | 2025 (12.8) | | 3759 (12.7) | 1418 (19.1) |
| Ischemic heart disease | 1713 (1.8) | | 1049 (2.0) | | 246 (1.6) | | 567 (1.9) | 236 (3.2) |
| Arrhythmias | 1112 (1.2) | | 712 (1.4) | | 191 (1.2) | | 352 (1.2) | 169 (2.3) |
| Hypertension | 3186 (3.4) | | 2446 (4.6) | | 692 (4.4) | | 1154 (3.9) | 600 (8.1) |
| Anemia | 552 (0.6) | | 1730 (3.3) | | 770 (4.9) | | 602 (2.0) | 358 (4.8) |
| Diabetes | 1887 (2.0) | | 1317 (2.5) | | 335 (2.1) | | 697 (2.4) | 285 (3.8) |
| Obesity | 1012 (1.1) | | 613 (1.2) | | 225 (1.4) | | 235 (0.8) | 153 (2.1) |
| Dyslipidemia | 1122 (1.2) | | 690 (1.3) | | 162 (1.0) | | 358 (1.2) | 170 (2.3) |
| Sleep problems | 1251 (1.3) | | 813 (1.5) | | 248 (1.6) | | 394 (1.3) | 171 (2.3) |
| Chronic kidney diseases | 203 (0.2) | | 273 (0.5) | | 76 (0.5) | | 120 (0.4) | 77 (1.0) |
| COPD ^e^ | 539 (0.6) | | 481 (0.9) | | 159 (1.0) | | 215 (0.7) | 107 (1.4) |
| Autoimmune diseases | 7621 (8.0) | | 6546 (12.4) | | 2509 (15.9) | | 2714 (9.2) | 1323 (17.8) |
| Follow-up time, years |  | |  | |  | |  |  |
| Median (IQR) | 13.7 (7.8-19.8) | | 13.4 (7.7-19.3) | | 13.9 (7.9-20.5) | | 14.2 (8.3-19.7) | 9.9 (5.9-15.8) |
| <1 | 1253 (1.3) | | 805 (1.5) | | 217 (1.4) | | 391 (1.3) | 197 (2.7) |
| 1-4 | 9495 (10.0) | | 5386 (10.2) | | 1560 (9.9) | | 2582 (8.7) | 1244 (16.8) |
| 5-9 | 22360 (23.5) | | 12606 (23.9) | | 3618 (22.9) | | 6666 (22.6) | 2322 (31.3) |
| 10-19 | 39003 (41.0) | | 21739 (41.2) | | 6225 (39.5) | | 12821 (43.4) | 2693 (36.3) |
| ≥20 | 23128 (24.3) | | 12225 (23.2) | | 4151 (26.3) | | 7107 (24.0) | 967 (13.0) |
| CD: Crohn’s disease; COPD: chronic obstructive pulmonary disease; CVD: cardiovascular diseases; IBD(-U): inflammatory bowel disease (unclassified); IQR: interquartile range; SD: standard deviation; UC: ulcerative colitis. | | | | | | | | |
| ^a^ Index date: date of IBD diagnosis for patients, and date of selection for their matched population references.  ^b^ Nordic countries: Sweden, Denmark, Finland, Norway, and Iceland. | | | | | | | | |
| ^c^ Defined as the number of healthcare visits between 2 years and 6 months before the index date.  ^d^ See **Table S4** for diseases' definitions.  ^e^ Only if the patient was diagnosed ≥ 40 years of age. | | | | | | | | |

| **Table S11** Incident heart failure in patients with IBD and their IBD-free full siblings | | | | | |
| --- | --- | --- | --- | --- | --- |
|  | No. of events (IR, per 10,000 person-years) | | IR difference (95%CI), per 10,000 person-years | HR (95%CI) | |
|  | Patients | References |  | Model 1 ^a^ | Model 2 ^b^ |
| Overall IBD | 1705 (22.5) | 2916 (21.0) | 1.5 (0.2 to 2.8) | 1.24 (1.16 to 1.32) | 1.10 (1.03 to 1.19) |
| CD | 525 (22.2) | 860 (20.0) | 2.2 (-0.1 to 4.5) | 1.28 (1.13 to 1.44) | 1.15 (1.00 to 1.31) |
| UC | 947 (21.7) | 1697 (21.1) | 0.6 (-1.1 to 2.3) | 1.18 (1.09 to 1.29) | 1.06 (0.96 to 1.16) |
| IBD-U | 233 (27.5) | 359 (22.8) | 4.7 (0.4 to 8.9) | 1.40 (1.17 to 1.68) | 1.25 (1.02 to 1.53) |
| CD: Crohn's disease; CI: confidence interval; HR: hazard ratio; IBD(-U): inflammatory bowel disease (unclassified); IR: incidence rate; UC: ulcerative colitis. | | | | | |
| ^a^ Conditioned on family identifier and adjusted for birth year, sex, county of residence, and calendar year. | | | | | |
| ^b^ Further adjusted for country of birth, educational attainment, number of healthcare visits, ischemic heart disease, arrhythmias, hypertension, anemia, dyslipidemia, diabetes, obesity, sleep problems, chronic kidney diseases, chronic obstructive pulmonary disease (only if diagnosed ≥ 40 years), and autoimmune diseases. | | | | | |

| **Table S12** Characteristics of incident heart failure at time of first diagnosis of heart failure in patients with IBD and their matched reference individuals | | |
| --- | --- | --- |
|  | Incident HF in IBD patients | Incident HF in reference individuals |
| N | 5582 | 20343 |
| Age at HF diagnosis, years |  |  |
| Mean ± SD | 74.8 ± 12.2 | 75.9 ± 11.8 |
| Median (IQR) | 76.6 (68.1-83.6) | 77.6 (68.9-84.6) |
| Female, n (%) | 2467 (44.2) | 8508 (41.8) |
| Comorbidities before HF diagnosis, n (%) |  |  |
| Ischemic heart disease | 2069 (37.1) | 6856 (33.7) |
| Myocardial infarction | 1179 (21.1) | 4133 (20.3) |
| Atrial fibrillation/flutter | 1569 (28.1) | 5677 (27.9) |
| Hypertension | 2682 (48.1) | 9218 (45.3) |
| Stroke | 824 (14.8) | 2973 (14.6) |
| Anaemia | 722 (12.9) | 1003 (4.9) |
| Dyslipidaemia | 656 (11.8) | 2631 (12.9) |
| Diabetes | 1212 (21.7) | 4038 (19.9) |
| Obesity | 270 (4.8) | 906 (4.5) |
| Chronic kidney diseases | 460 (8.2) | 1111 (5.5) |
| COPD | 775 (13.9) | 2044 (10.1) |
| COPD: chronic obstructive pulmonary disease; HF: heart failure; IQR: interquartile range; SD: standard deviation. | | |


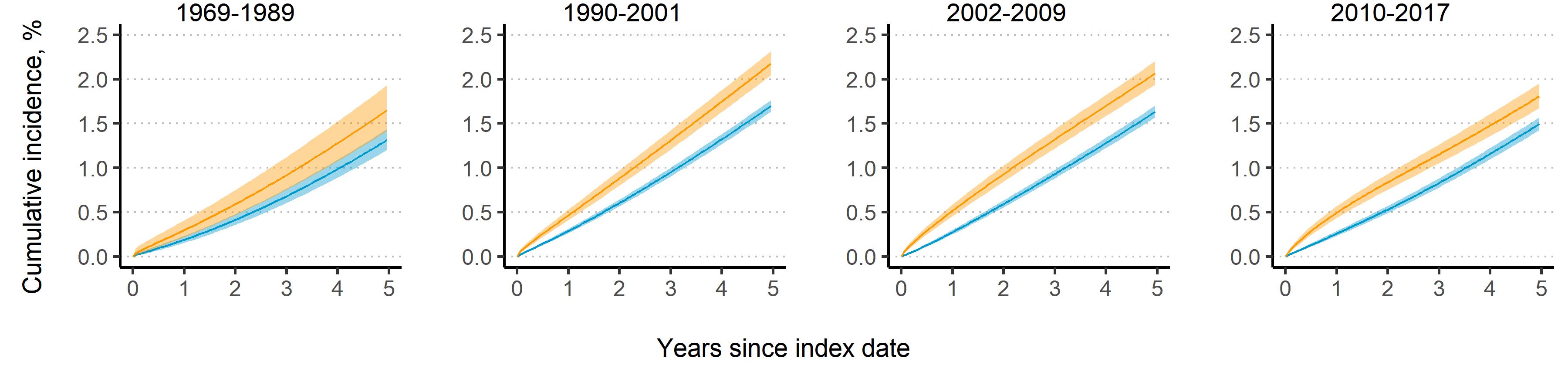


**Figure S1** Standardized cumulative incidence with 95% confidence interval (CI) for heart failure, stratified by calendar period at index date.

The standardized cumulative incidence was estimated from the adjusted flexible parametric survival model. Yellow: patients with inflammatory bowel disease; blue: the reference individuals from the general population.


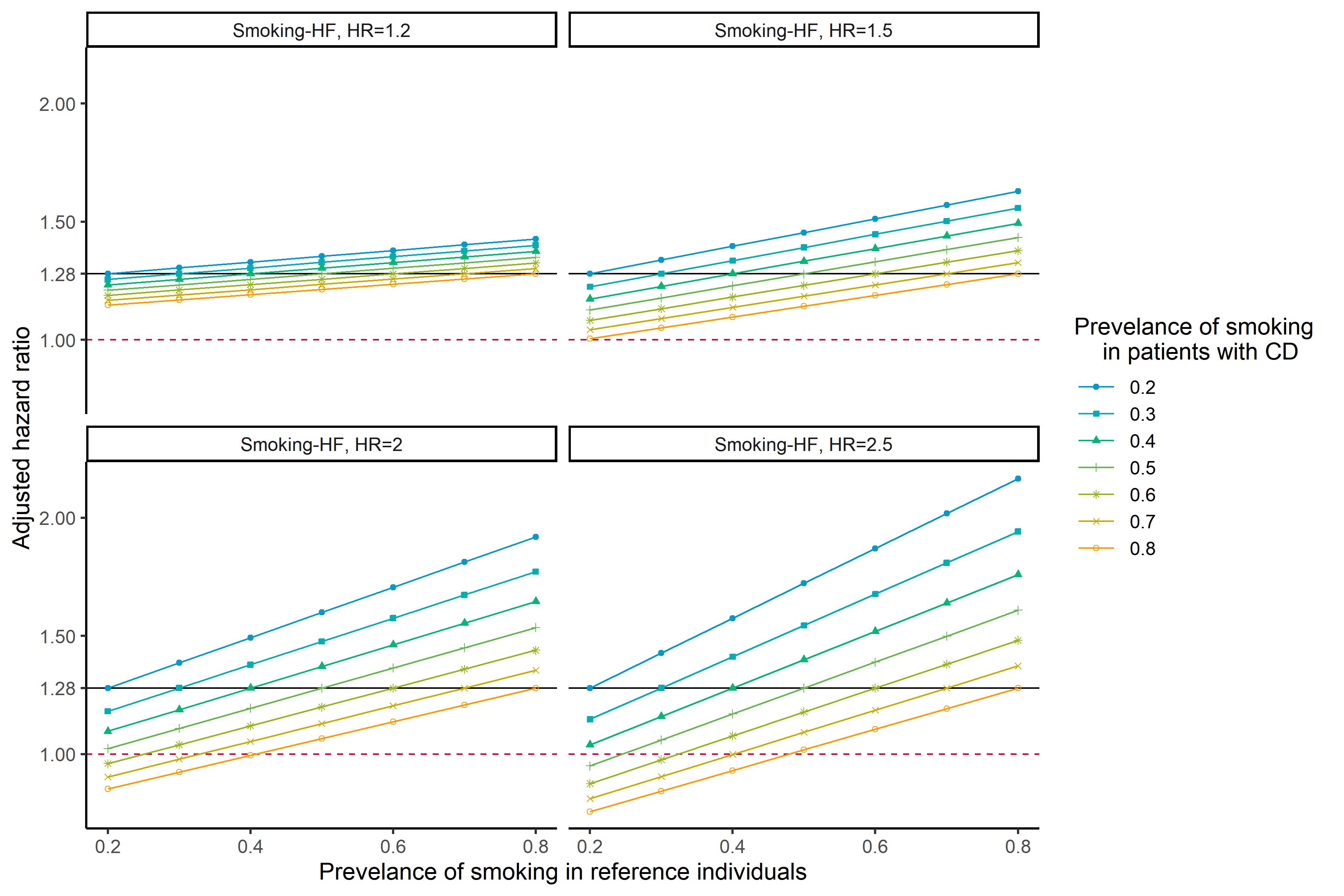


**Figure S2** Sensitivity analysis of unmeasured confounding for the association of CD with heart failure due to smoking.

The figure illustrates the estimated hazard ratio (HR, on the y-axis) that would have been observed if the prevalence of smoking was measured in both CD patients and their matched reference individuals and smoking had an assumed association with heart failure (HF), using R package tipr^8^. The solid black line is the observed association between CD and HF in the sample (aHR=1.28), while the red dotted line denotes the threshold for a null association.

Explanation (lower right panel): smoking with an association with HF as strong as a HR of 2.5 would need to have an around 30% difference in prevalence between patients with CD and reference individuals (e.g., 80% in patients with CD and ~50% in the reference individuals) to make the estimated HR (y-axis) below one. However, smoking cannot fully explain away our observed association between CD and HF for two main reasons. First, although previous evidence has suggested that smoking was positively associated with HF (summary RR=1.75 for current smokers, summary RR=1.16 for former smokers, summary RR=1.41 per 10 cigarettes per day, and summary RR=1.08 per 10 pack-years in former smokers^9^), all these summary RRs are smaller than two. Second, according to a study from Sweden (study period: 2005-2009), about 30.7% patients with CD were either current or former smokers^10^; although in Sweden the prevalence of daily smoking has decreased from 14% in 2006 to 6% in 2021 among individuals aged 16-84 years, the use of daily snuff (a smokeless tobacco product) in the same population still remains high (20% in men and 6% in women in 2021) (<https://www.folkhalsomyndigheten.se/the-public-health-agency-of-sweden/living-conditions-and-lifestyle/>). Therefore, the difference of smoking prevalence between patients with CD and their matched reference individuals is unlikely to be larger than 30%.

On the other hand, since smoking was inversely associated with UC (odds ratio=0.58 for current smoker^11^) but positively associated with heart failure, not adjusting for smoking would bias the association between UC and HF toward null.

**References**

1. Prasada S, Rivera A, Nishtala A, Pawlowski AE, Sinha A, Bundy JD, et al. Differential Associations of Chronic Inflammatory Diseases With Incident Heart Failure. JACC Heart Fail. 2020;8(6):489-98.

2. Aniwan S, Pardi DS, Tremaine WJ, Loftus EV. Increased Risk of Acute Myocardial Infarction and Heart Failure in Patients With Inflammatory Bowel Diseases. Clinical Gastroenterology and Hepatology. 2018;16(10):1607-15.e1.

3. Kristensen SL, Ahlehoff O, Lindhardsen J, Erichsen R, Lamberts M, Khalid U, et al. Inflammatory Bowel Disease Is Associated With an Increased Risk of Hospitalization for Heart Failure. Circulation: Heart Failure. 2014;7(5):717-22.

4. Forss A, Clements M, Bergman D, Roelstraete B, Kaplan G, Gilaad, Myrelid P, et al. A nationwide cohort study of the incidence of inflammatory bowel disease in Sweden from 1990 to 2014. Alimentary Pharmacology & Therapeutics. 2022;55(6):691-9.

5. Mouratidou N, Malmborg P, Järås J, Sigurdsson V, Sandström O, Fagerberg UL, et al. Identification of Childhood-Onset Inflammatory Bowel Disease in Swedish Healthcare Registers: A Validation Study. Clinical Epidemiology. 2022;Volume 14:591-600.

6. Olén O, Erichsen R, Sachs MC, Pedersen L, Halfvarson J, Askling J, et al. Colorectal cancer in ulcerative colitis: a Scandinavian population-based cohort study. The Lancet. 2020;395(10218):123-31.

7. Ludvigsson JF, Inghammar M, Ekberg M, Egesten A. A nationwide cohort study of the risk of chronic obstructive pulmonary disease in coeliac disease. Journal of Internal Medicine. 2012;271(5):481-9.

8. D’Agostino McGowan L. Sensitivity Analyses for Unmeasured Confounders. Current Epidemiology Reports. 2022;9(4):361-75.

9. Aune D, Schlesinger S, Norat T, Riboli E. Tobacco smoking and the risk of heart failure: A systematic review and meta-analysis of prospective studies. Eur J Prev Cardiol. 2019;26(3):279-88.

10. Sjoberg D, Holmstrom T, Larsson M, Nielsen AL, Holmquist L, Ekbom A, et al. Incidence and clinical course of Crohn's disease during the first year - results from the IBD Cohort of the Uppsala Region (ICURE) of Sweden 2005-2009. J Crohns Colitis. 2014;8(3):215-22.

11. Piovani D, Danese S, Peyrin-Biroulet L, Nikolopoulos GK, Lytras T, Bonovas S. Environmental Risk Factors for Inflammatory Bowel Diseases: An Umbrella Review of Meta-analyses. Gastroenterology. 2019;157(3):647-59 e4.
